# Impact of sample history and solvent effects on pathway control in the supramolecular polymerisation of Au(i)-metallopeptide amphiphiles†

**DOI:** 10.1039/d3py00053b

**Published:** 2023-04-06

**Authors:** Marius Thomas, Vanessa Lewe, Jonas Kölsch, Moritz Urschbach, Jessica Erlenbusch, Oliver Sven Stach, Pol Besenius

**Affiliations:** a Department of Chemistry, Johannes Gutenberg-University Mainz Duesbergweg 10-14 D-55128 Mainz Germany besenius@uni-mainz.de; b Graduate School of Materials Science in Mainz Staudingerweg 9 D-55128 Mainz Germany

## Abstract

We investigate the kinetics of the supramolecular polymerisation of an Au(i)-metallopeptide amphiphile that assembles into exceptionally long and rigid nanofibers. We developed a precise preparation protocol to measure the concentration dependent assembly kinetics which elucidated a nucleation–elongation dominated supramolecular polymerisation process. We show striking differences in the assembly behavior and morphology in aqueous media, even at organic solvent contents as low as 1 vol%, compared to pure buffer.

## Abstract 2

We investigate the kinetics of the supramolecular polymerisation of an Au(i)-metallopeptide amphiphile that assembles into exceptionally long and rigid nanofibers.

## Graphical abstract

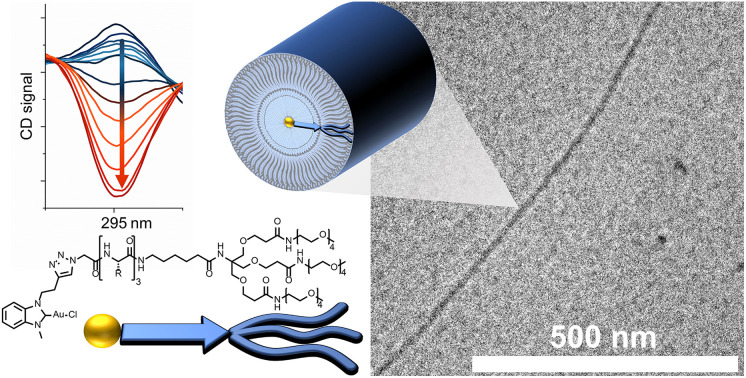

Embedding non-covalent interaction motifs into molecular subunits is an extensively used strategy to design and synthesise supramolecular polymers, with a hierarchical order in one-, two- or three-dimensional architectures.^1–4^ These have a considerable impact in the field of self-assembled molecular materials, and the evaluation of their mechanical, optoelectronic or biological functions.^5^ Similar to covalent polymer materials, mechanistic investigations are crucial for polymer syntheses and preparation protocols, which have direct impact on the degree of polymerisation, dispersity or polymer microstructures.^6–8^ Compared to the sophisticated covalent (co)polymerisation protocols, supramolecular polymer methodologies have neglected kinetic investigations to a large extent^9–11^ and the impact of pathway complexity has only fairly recently been recognised in the construction of supramolecular systems. An early example from the Rybtchinski group investigated the role of temperature and kinetic profiles in the supramolecular polymerisation of naphthalene diimide-Pt(ii) amphiphiles in H_2_O/THF mixtures, which gave rise to a rich energy landscape and multiple kinetically stable intermediates en route to the most stable supramolecular polymer fibres.^12^ The Meijer lab reported hydrogen bonded oligo-phenylene vinylenes in apolar organic solvents, with off-pathway *P*-helical polymers that consume free monomers and slow down the formation of thermodynamically more stable *M*-helical supramolecular polymers.^13^ De Cola and coworkers used luminescent Pt(ii)-complexes, with intricate behaviour in H_2_O/alcohol mixtures.^14^ Off-pathway blue emissive complexes are kinetically favoured but over time consumed to form thermodynamically more stable red-emissive stacks. The supramolecular polymer field has generally accepted the notion that polymorphism is indeed possible if multiple pathways compete,^15–18^ and that an evaluated energy landscape with their respective pathways is only applicable to a specific set of experimental conditions.^17^ Increasing efforts in elucidating complex assembly pathways have been reported using temperature, concentration or time as variable parameters.^19,20^ In addition a considerable number of studies investigated the impact of the solvent and solvent composition in supramolecular polymerisations.^21–26^

However, in our opinion the preparation of molecular precursors with respect to purification and especially isolation conditions and the sample preparation protocol are aspects that have generally been overlooked.^27^ In this body of work, we therefore specifically focus on the impact of isolation conditions on supramolecular peptide monomers and the impact of the sample preparation protocol on the mechanisms and kinetics of supramolecular polymerization. We chose an Au(i)-metallopeptide amphiphile, with solubilising dendritic oligoethylene glycol chains, and an acidic tetrapeptide domain. It is well suited to investigate sample history and solvent effects on pathway control in supramolecular polymerization, given its high solubility in water and reversibly charged state to tune the driving force for supramolecular polymerization under buffered conditions.

The Au(i)-metallopeptide amphiphile 1 (Fig. 1) was synthesised from an alkyne functionalised hydrophobic N-heterocyclic carbene (NHC)–Au(i) complex 2 and an azide modified amphiphilic peptide building block 4*via* Cu(i)-catalysed azide–alkyne cycloaddition (CuAAC). Using an aminohexanoic acid (Ahx) spacer, the hydrophobic peptide domain was linked to a hydrophilic Newkome-like dendron bearing a tetraethylene glycol unit which provides high water solubility (Fig. 1). Unlike previous reports, the dendritic unit does not bear charges in the periphery,^28^ and repulsive Coulomb interactions are solely embedded in the peptide domain. As the purity of the substrate plays a crucial role in peptide self-assembly kinetics,^29^ compound 1 was purified using semi-preparative HPLC to remove any impurity in the synthesis process that could be structurally related to the substrate studied and could potentially act as a binder or interaction partner during the self-assembly process. Since the Au(i)NHC complex is not stable under highly acidic conditions, 1 mM NH_4_HCO_3_ buffer at pH 7.9 was used to ensure a slightly basic environment and the deprotonation of the glutamic acid residue (Fig. 1), which in turn suppresses aggregation under aqueous conditions during chromatography due to repulsive Coulomb interactions of the deprotonated glutamic acid side chain.

**Fig. 1:**
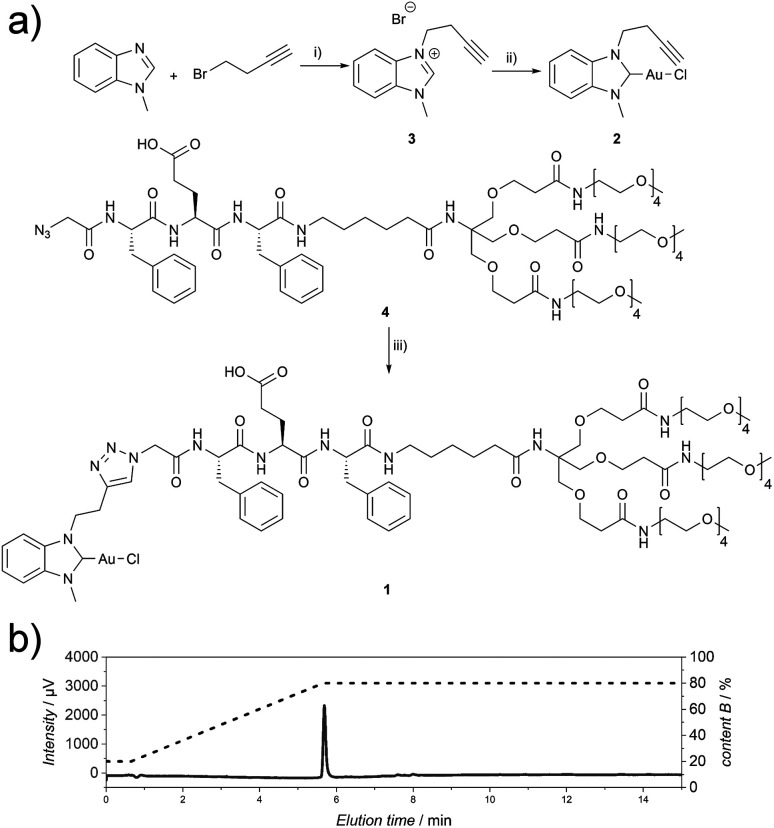
(a) Synthetic route for the synthesis of compound 1. (i) 1-Methyl-1*H*-benzo[*d*]-imidazole (1.0 eq.), 4-bromobut-1-yne (1.5 eq.), toluene, 110 °C, 72 h; (ii) (1) 3 (1.0 eq.), Ag_2_O (0.6 eq.), DCM, r.t., 68 h, (2) Au(i)Cl(tht) (1.0 eq.), NaCl (1.5 eq.), r.t., 2.5 h; (iii) 2 (1.1 eq.), 4 (1.0 eq.), CuSO_4_·5H_2_O (0.2 eq.), TBTA (0.2 eq.), sodium l-ascorbate (0.2 eq.), THF : H_2_O, 1 : 1, 45 °C, 7 h. (b) Analytical HPLC chromatogram (215 nm) of 1 (CH_3_CN : H_2_O gradient, 1 mM NH_4_HCO_3_).

To investigate the structural change during the supramolecular polymerisation of compound 1, circular dichroism (CD) spectroscopy was used (full sample preparation protocols are presented in the ESI†). CD spectroscopy is a valuable tool for the characterisation of self-assembly processes, as it gives rise to specific signals from chromophores in ordered structures like secondary structures of peptides or exciton bands of spatially arranged chromophores. We studied the pH responsiveness of the system by dissolving compound 1 in a CH_3_CN : H_2_O (1 : 1, *V* : *V*) mixture to yield a solution of pH 6, which is indicative of an aggregated structure (Fig. S1†). After adjusting the solution to pH 12 no significant CD signal can be seen at a basic pH value due to the repulsive Coulomb interaction of the glutamic acid residues in an acetonitrile water mixture and reduced driving force for supramolecular polymerisation. By measuring CD spectra after lowering the pH value the appearance of different CD signals can be observed, which we assign to the formation of ordered supramolecular structures (positive CD bands at *λ* = 295 nm and 286 nm and negative bands at *λ* = 298 nm, 282 nm, 275 nm, 240 nm and 227 nm, Fig. 2a). By plotting the intensity of the CD signal at a wavelength of *λ* = 295 nm a transition is observed at around pH = 6 (Fig. 2b), which correlates with the apparent p*K*_a_ value in the glutamic acid side chain. This increase with respect to values from isolated molecularly dissolved glutamic carboxylic groups is due to the strong driving force for self-assembly in amphiphilic oligopeptides.^30–32^ Surprisingly, however, the characteristic CD bands do not disappear under basic aqueous conditions and the absence of CH_3_CN (see ESI Fig. S2†). We suspected that in the absence of an organic solvent the supramolecular structure formation could not be shifted to molecularly dissolved species, highlighting either their high thermodynamic stability or an intrinsic kinetic barrier for the reverse reaction and disassembly process. This observation confirms the importance of choosing suitable solvent compositions for the sample purification and isolation steps, in order to operate under conditions where the amphiphile is molecularly dissolved. Molecular interactions with remaining impurities and co-elution have to be avoided. Furthermore, solvent composition is key during the sample preparation to make sure that the molecular amphiphile is not pre-aggregated prior to dissolution in a medium that induces self-assembly like aqueous buffers, where the pre-aggregates potentially act as seeds and nucleate the supramolecular polymerisation.

**Fig. 2:**
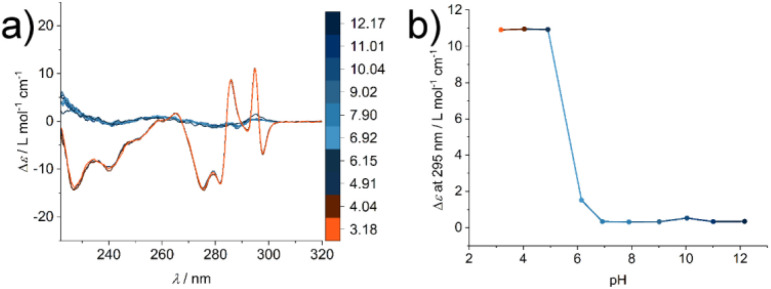
(a) CD spectra of a 400 μM solution of compound 1 in CH_3_CN : H_2_O (1 : 1) at different pH values (color scale): beginning at pH 12.17, the pH value was adjusted to 3.18. (b) Specific ellipticity values monitored at *λ* = 295 nm show the transition from an unassembled state under basic conditions to an aggregated state at acidic pH.

After purification *via* HPLC chromatography (1 mM NH_4_HCO_3_, pH 7.9) and lyophilisation of compound 1 from the CH_3_CN : H_2_O mixture, the isolated material was redissolved in 10 mM phosphate buffer at pH 7.4 and room temperature. CD spectra were measured immediately at different time points. To our surprise the self-assembly process was fast and even at a temperature of 5 °C early time points could not be resolved on the timescale of the experiment with a degree of polymerisation *α* = 0.52 at the first data point (Fig. S3†). The fast assembly kinetics suggest a seeded growth into supramolecular assemblies, most likely one-dimensional fibres. We hypothesized that pre-aggregates exist in this sample preparation method, probably forming in different solvent compositions during the lyophilisation process.

In addition, the sample was characterised in solution in dimethyl sulfoxide (DMSO) as a strong H-bond acceptor and hexafluoroisopropanol (HFIP) as a strong H-bond donor. Both CD spectra are indicative of molecularly dissolved species in dilute solutions (Fig. 3). Using a solution of molecularly dissolved Au(i)-metallopeptide amphiphile 1 in HFIP further kinetic experiments were performed, in which a concentrated solution of the monomer was diluted hundredfold in 10 mM phosphate buffer at pH 7.4 at 5 °C (Fig. S4†). The self-assembled structure shows a slightly different CD spectrum (positive CD bands at *λ* = 294 nm and 285 nm and a strong negative band at *λ* = 297 nm, and negative bands at *λ* = 282 nm, 276 nm, 241 nm and 227 nm), while the formation kinetics was very fast and could not be resolved again on the timescale of the experiment with *α* = 0.88 at the first data point, similar to the results obtained using isolated molecular amphiphiles from CH_3_CN : H_2_O mixtures. This indicates that the influence of HFIP content (1 vol%) cannot be neglected. We performed morphological investigations using transmission electron microscopy (TEM), which clearly show the formation of individual fibres with an average length of *L*_n_ = 111 nm (*L*_w_ = 161 nm, *σ* = 75 nm *Đ* = 1.45, *n* = 691), which do not bundle or cluster (Fig. S5†). It is noted that fluorinated solvents like hexafluoroisopropanol or trifluoroethanol (TFE) are indeed able to stabilise the formation of peptide secondary structures, particularly in solvent mixtures with water. The enhanced stability is a result of favourable solvation of exposed hydrophobic peptide side chains and strengthening of hydrogen bonding, whereas alcohol clustering and solvent mixture microheterogeneities were suggested to enhance these effects further.^33–35^ In contrast, lyophilisation of Au(i)-metallopeptide amphiphile 1 from DMSO and dissolving the molecule in 10 mM phosphate buffer gave rise to concentration dependent self-assembly kinetics (Fig. S6†), which are significantly slower than those of previous preparation protocols or solvent conditions. The obtained half times of polymerisation *t*_50_ range from 498 min at 200 μM to 126 min at 800 μM. To confirm the morphology of the produced supramolecular polymers, cryogenic transmission electron microscopy (cryoTEM) experiments were performed (Fig. 4). Rigid μm-long fibres are clearly observed after four days of equilibration, with an absolute length of >1 μm and length distribution that cannot be resolved. In addition, a slightly different altered CD spectrum was obtained with negative bands at *λ* = 295 nm and 286 nm and positive bands at 282 nm and 275 nm (Fig. S6†). Under the assumption of a molecularly dissolved species in DMSO and a completely polymerised state at saturation of the CD signal, the monitored CD signal intensity at a wavelength of *λ* = 295 nm was normalised. The resulting time dependent degree of polymerisation *α*(*t*) was plotted in a concentration dependent manner (Fig. 5a). It can be seen that the supramolecular polymer formation accelerates with increasing total monomer concentration (Fig. 5b), which is in accordance with general kinetic considerations.

**Fig. 3:**
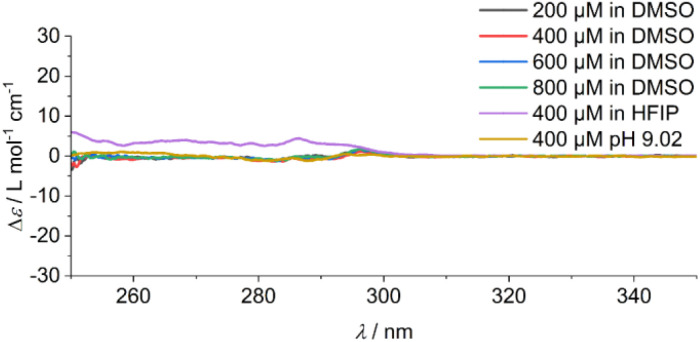
CD spectra of solutions of Au(i)-metallopeptide amphiphile 1 in DMSO at the measured concentrations, and the spectra of a 400 μM solution in HFIP and in a CH_3_CN : H_2_O (1 : 1) solvent mixture at pH 9.02. All spectra are indicative of a disassembled and molecularly dissolved state.

**Fig. 4:**
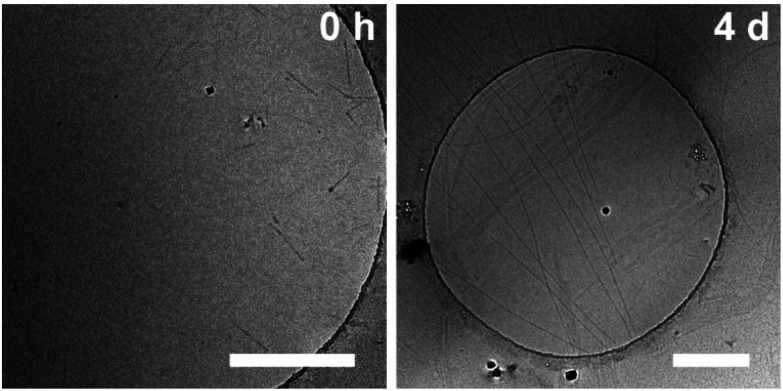
cryo-TEM micrographs of Au(i)-metallopeptide amphiphile 1 (lyophilized from DMSO) in 10 mM phosphate buffer at pH 7.4 straight after sample preparation (0 h, left) and after full assembly (4 d, right). Scale bars represent 500 nm.

**Fig. 5:**
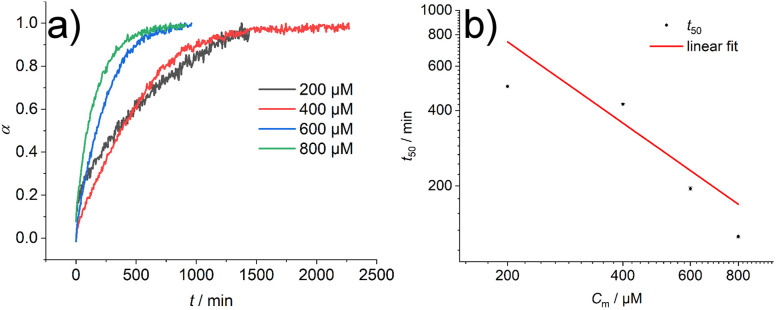
(a) Degree of polymerisation *α* for the kinetic measurement of compound 1 lyophilized from DMSO and dissolved in 10 mM phosphate buffer at pH 7.4 at 5 °C. (b) Double logarithmic plot of the half times *t*_50_ of the self-assembly of compound 1 at different monomer concentrations *C*_m_. Linear fit of the data points shows a slope of γ = −1.07.

Rather than using the described preparation protocol *via* the complete removal of DMSO by lyophilisation, a different protocol was further evaluated. Using a solution of molecularly dissolved compound 1 in DMSO and hundredfold dilution in 10 mM phosphate buffer (pH 7.4) at 5 °C the self-assembly kinetics could be investigated again (Fig. S7–S9†). However, rather unexpectedly the concentration dependent supramolecular polymerisation this time gives rise to a profile that seems nearly independent of the monomer concentration (characteristic half times on the order of 132 min to 269 min), again corroborating the fact that the remaining traces of solvents are not neglectable when evaluating the dynamics of supramolecular polymers in water. When comparing the obtained CD spectra of the two different kinetic experiments two sets of initial CD signals were found, indicating the presence of different initial aggregates, and both lead to the same final structure (further discussion is presented in the ESI†). Morphological studies using TEM show very long and rigid fibres with an absolute length of >1 μm that cannot be resolved, which also show a tendency to cluster into bundles of fibres (Fig. S10†).

To gain first insights into the mechanisms involved in the formation of the one-dimensional supramolecular polymers, we investigated the scaling behaviour of the concentration dependent polymerisation kinetics of compound 1 without DMSO content. The half time of polymerisation *t*_50_ is defined as the time point at which half of the final aggregate concentration is reached.^13,36^ Using a double logarithmic plot of these halftimes against the monomer concentration *C*_m_ (Fig. 5b), the scaling exponent *γ* can be obtained using a linear fit from the power law *t*_50_ ∝ *C*^*γ*^_m_^36^ The slope of *γ* = −1.07 is clearly indicative of a nucleation–elongation dominated mechanism, which is a hallmark of ordered supramolecular polymers.^37^

## Conclusions

We have shown the supramolecular polymerisation of an Au(i) metallopeptide amphiphile from a molecularly dissolved state into exceptionally long and rigid one-dimensional nanofibers. In particular, we highlight the role of the choice of solvent and solvent composition in the sample preparation and tuning of experimental conditions when evaluating the kinetics of supramolecular polymerisation in aqueous environments. Even small quantities as low as 1 vol% organic content can heavily influence the kinetic behaviour of the supramolecular polymerisation or even the yielded morphologies, such as the supramolecular polymer length. The impact of monomer purification, isolation and preparation on nucleated supramolecular polymerisation pathways is considerable and cannot be neglected.

By developing a precise sample preparation protocol, the supramolecular polymerisation kinetics could be measured. Concentration dependent half times *t*_50_ between 498 min and 126 min were measured in pure buffer by directly dissolving dry monomer 1, which was isolated and stored by lyophilisation from a molecularly dissolved state in DMSO. The scaling exponent from these kinetic experiments suggests a nucleation–elongation dominated mechanism for the polymerisation of monomer 1. On the other hand, the measured supramolecular polymerisation kinetics in buffer with organic solvent additives, *e.g.* 1 vol% HFIP or 1 vol% DMSO, could not be resolved on the timescale of the experiment with a degree of polymerisation of >50% at the first recorded data after the dissolution of the monomer (<2 min). The latter relied on selective solvent techniques and hundredfold dilution of a concentrated monomer solution from DMSO or HFIP in buffer. These were nearly two orders of magnitude faster and suggest that the presence of organic solvent clustering and solvent mixture microheterogeneities significantly increase the supramolecular polymerisation. We therefore issue a cautionary note on mechanistic supramolecular polymerisation studies in aqueous environments involving solvent mixtures, especially when extrapolating thermodynamic and kinetic parameters for purely aqueous buffers. Particularly, the role of solvents, solvent additives, and trace impurities is not neglectable^38^ and since they influence the kinetics of the system, the supramolecular assembly protocol has to be adapted accordingly. Our findings show similarities to crystallisation phenomena in molecular systems and aggregation processes of proteins.^18,39–41^

## Author contributions

M. T. performed the synthesis and kinetic experiments, analysed the data, prepared the figures and wrote the manuscript. V. L. and J. K. performed the preliminary experiments. M. U. and J. E. performed TEM and cryoTEM. O. S. S. provided materials for the synthesis and P. B. conceptualised and supervised the project and wrote the manuscript.

## Conflicts of interest

There are no conflicts to declare.

## Supplementary Material

PY-014-D3PY00053B-s001
