# Supplementary material for: Impact of sample history and solvent effects on pathway control in the supramolecular polymerisation of Au(i)-metallopeptide amphiphiles
Source: Polym Chem. 2023 Apr 6;14(16):1888–92. doi: 10.1039/d3py00053b (PMC10127225; doi:10.1039/d3py00053b)
Supplement: PY-014-D3PY00053B-s001 [file PY-014-D3PY00053B-s001.pdf]

Electronic Supplementary Material (ESI) for Polymer Chemistry.
This journal is © The Royal Society of Chemistry 2023

Electronic Supplementary Information

1 Additional Experimental Data

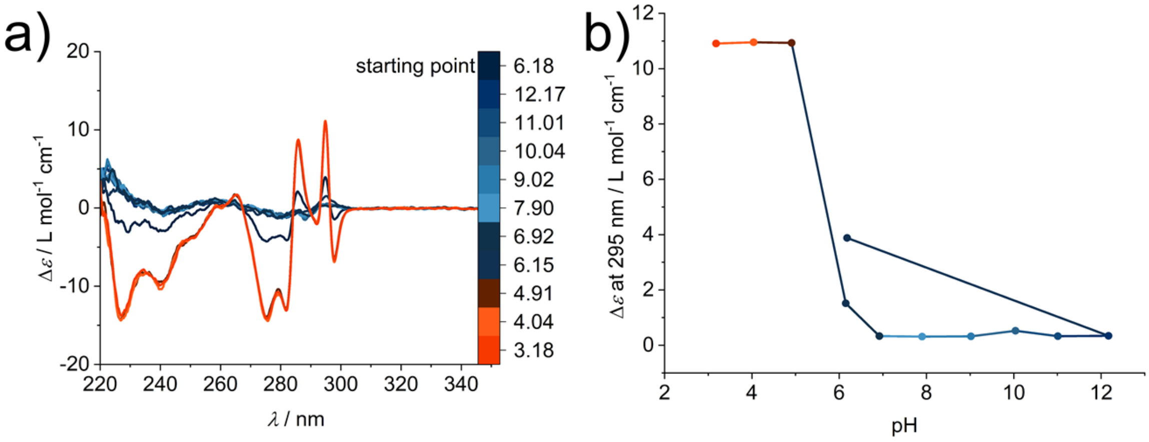

Figure S1. CD spectra of 400 μM solution of compound **1** in MeCN:H_2O (1:1, *v:v*) at different pH values. pH 6.18 results from just dissolving compound **1** in MeCN:H_2O (1:1, *v:v*) and marks the starting point. The pH was adjusted to 12.17 and then lowered stepwise to pH 3.18. b) CD-signal of compound **1** in MeCN:H_2O (1:1, *v:v*) at the wavelength $\lambda = 295\ nm$. At pH values above 7 the aggregates in MeCN:H_2O (1:1,*v:v*) fall apart because of Coulomb repulsion of the deprotonated glutamic acid residue. At lower pH the glutamic acid residue starts to get protonated and renders the molecules uncharged which promotes self-assembly.

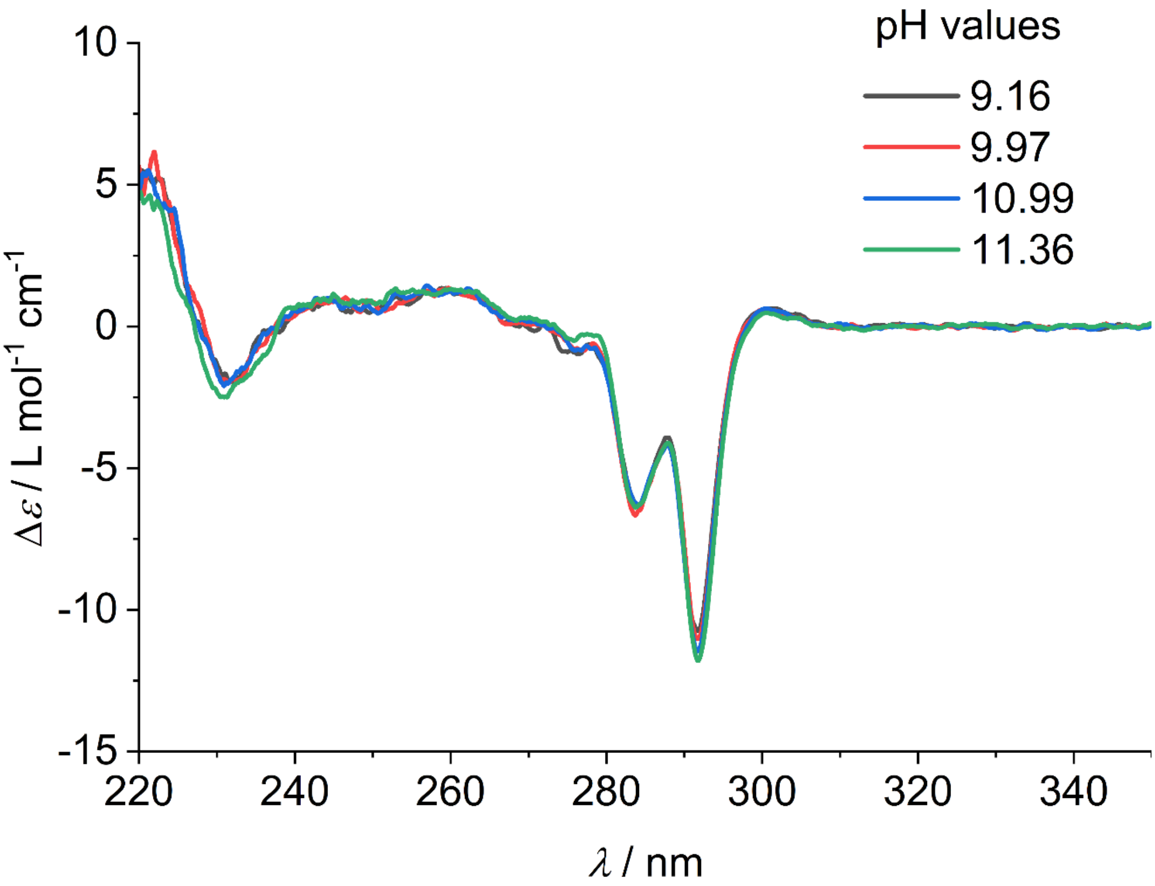

Figure S2. CD spectra of 400 μM solution of compound **1** in H_2O at different pH values. The CD-signal does not change with increase of the pH value. In the absence of an organic solvent the structure shows no pH responsiveness.

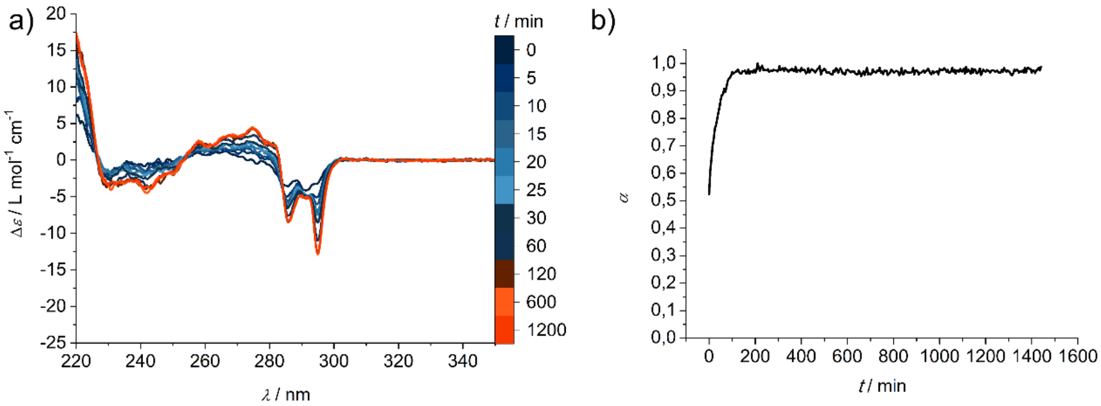

Figure S3. Kinetic CD measurement of compound **1** in 10 mM phosphate buffer at pH 7.4 at 5 °C dissolved from a lyophilized sample from MeCN:H_2O (1:1, *v*:*v*) at 400 μM concentration. b) degree of aggregation α with time t. The self-assembly shows surprisingly fast kinetics and cannot be resolved with the used experiment.

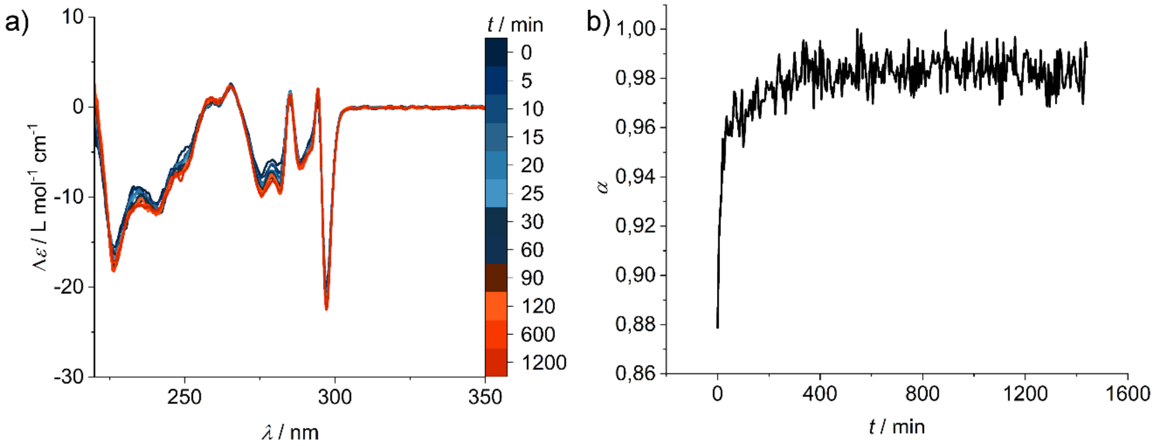

Figure S4. a) Kinetic CD measurement of compound **1** in 10 mM phosphate buffer at pH 7.4 at 5 °C with 1% HFIP at 400 µM concentration. b) degree of aggregation α with time t. The self-assembly shows surprisingly fast kinetics and cannot be resolved with the used experiment.

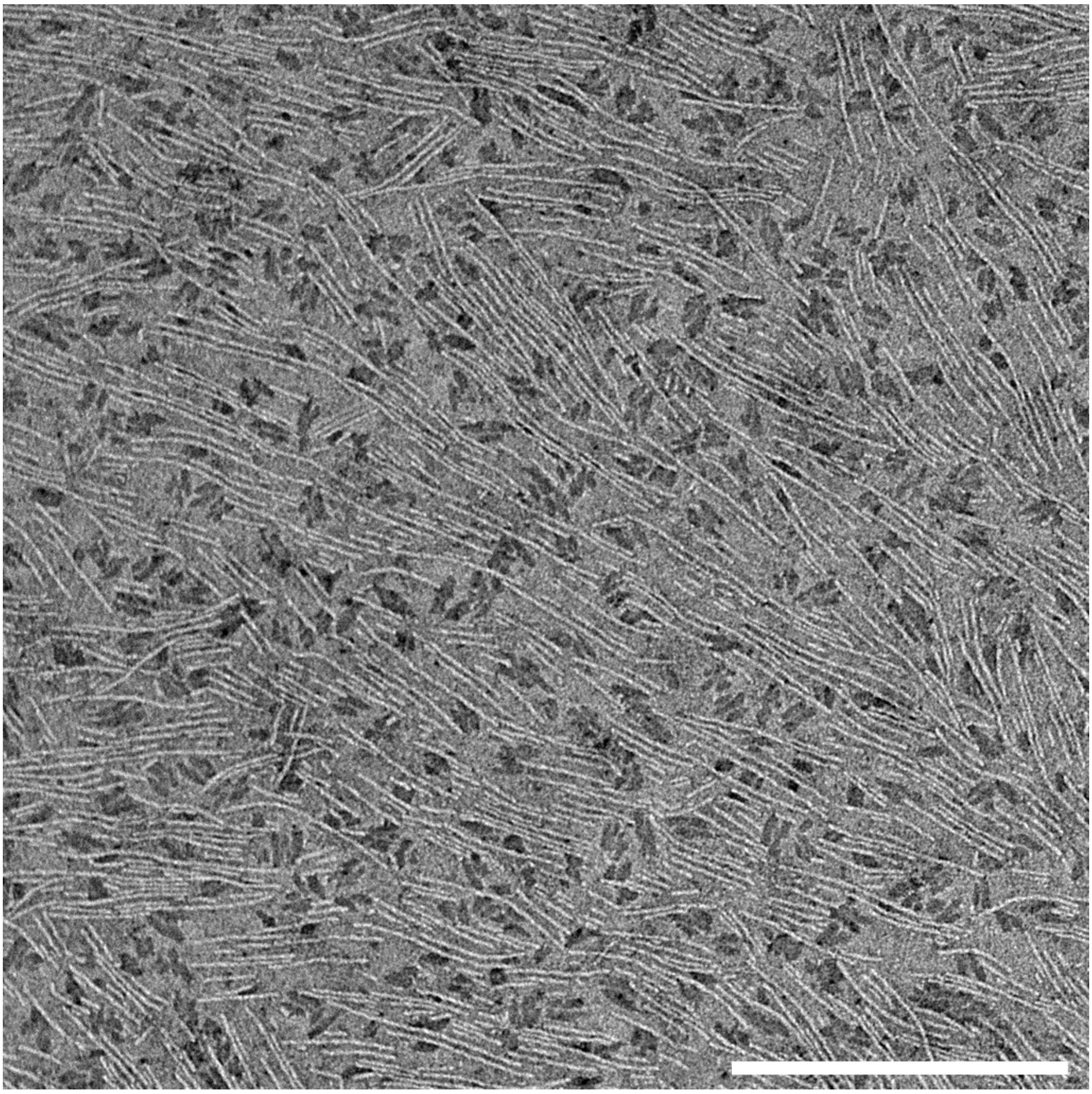

Figure S5. TEM micrograph of a 400 μM solution of compound **1** in 10 mM phosphate buffer with 1 vol% HFIP content at pH 7.4; scale bar represents 500 nm. The measured number average fibre length is $L_n = 111\ nm$, the weighted average fibre length $L_w = 161\ nm$, and a dispersity $Đ = 1.45, n = 691$). The number of measured fibres is $n = 691$ and the standard deviation $\sigma = 75\ nm$.

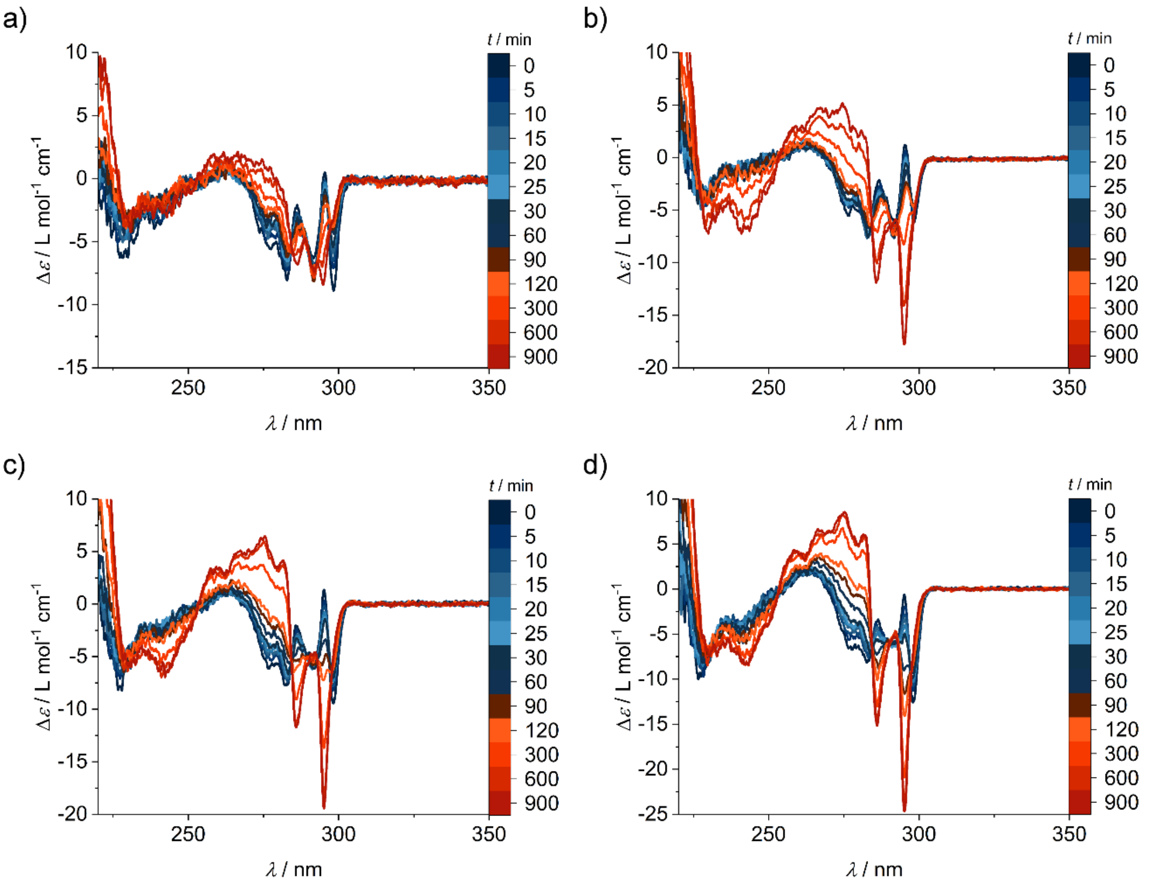

Figure S6. Kinetic CD measurements of compound **1** (lyophilised from DMSO) in 10 mM phosphate buffer at pH 7.4 at 5 °C at a) 200 µM, b) 400 µM, c) 600 µM and d) 800 µM concentration. The color scale shows different time points at which the measurement was recorded. The formation of the negative CD signal at $\lambda = 295\ nm$ can be followed (dark blue to red).

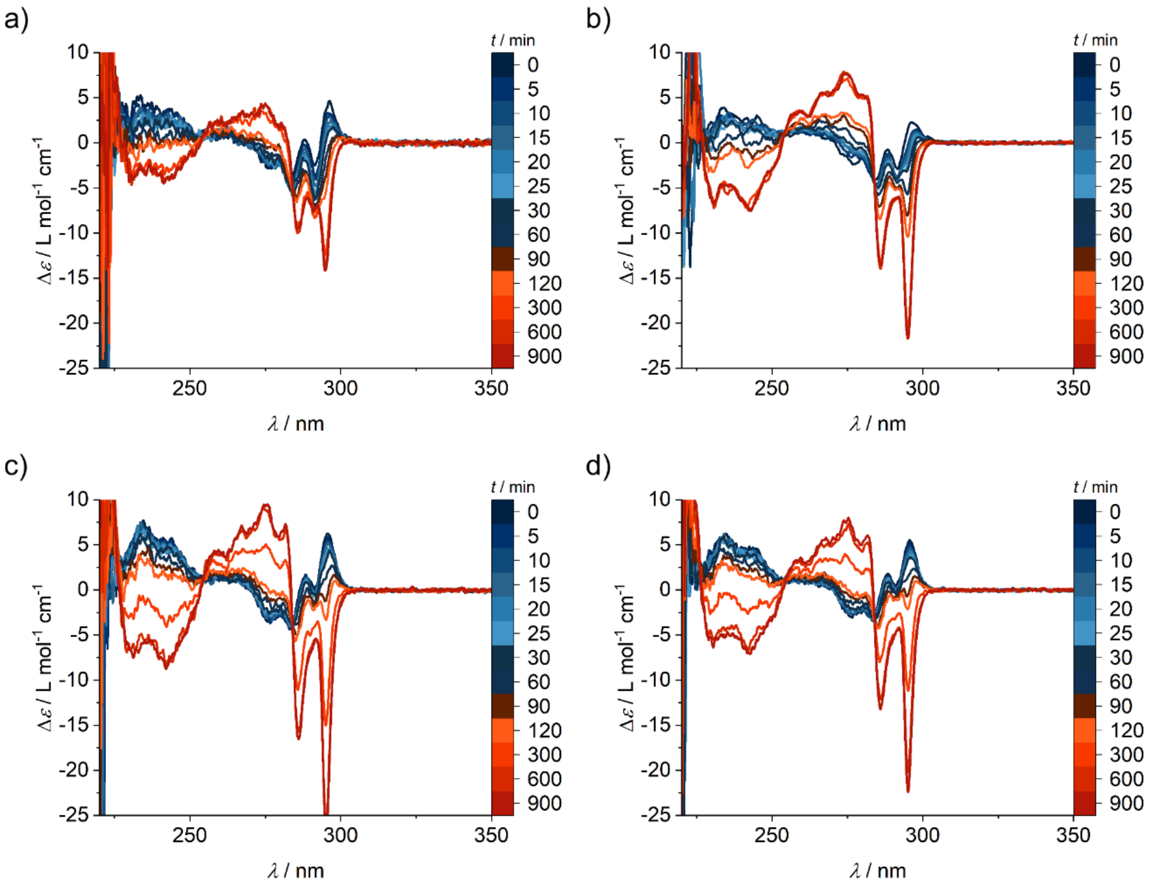

Figure S7. Kinetic CD measurements of compound **1** in 10 mM phosphate buffer with 1 vol% DMSO content at pH 7.4 at 5 °C at a) 200 µM, b) 400 µM, c) 600 µM and d) 800 µM concentration. The color scale shows different time points at which the measurement was recorded. The formation of the negative CD signal at $\lambda = 295\ nm$ can be followed (dark blue to red).

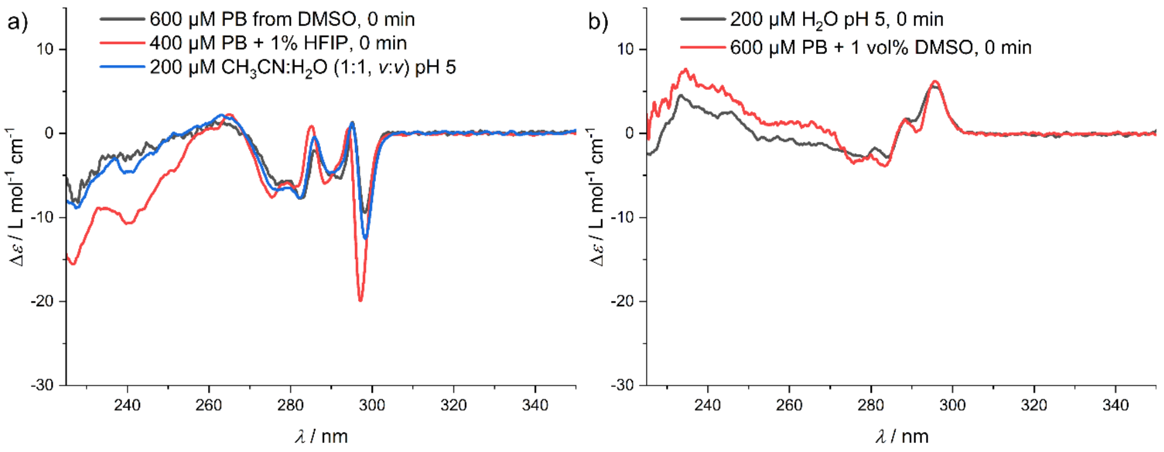

Figure S8. First recorded CD spectra of compound **1** after different sample preparation conditions with matching CD signals. a) compound **1** (lyophilised from DMSO) in 10 mM phosphate buffer at pH 7.4 at 5 °C at 600 µM concentration (black line), compound **1** in 10 mM phosphate buffer with 1 vol% HFIP content at pH 7.4 at 5 °C at 400 µM concentration (red line) and compound **1** in H_2O at pH 5 at 200 µM concentration with a 200 µM solution of compound **1** in CH_3CN added to the solution to yield a 200 µM solution in CH_3CN:H_2O (1:1, *v*:*v*) at pH 5 (blue line). b) compound **1** in

H_2O at pH 5 at 200 µM concentration after adjusting the pH from pH 12 to 5 (black line) and compound **1** in 10 mM phosphate buffer with 1 vol% DMSO content at pH 7.4 at 5 °C at 600 µM concentration (red line).

We found two different sets of initial signals in the first measured CD spectra for the starting points of the supramolecular polymerization of compound **1** in buffered solution under different preparation conditions. Compound **1** lyophilised from DMSO and dissolved in 10 mM phosphate buffer at pH 7.4 shows negative bands at $\lambda = 298\ nm, 292\ nm, 282\ nm$ and $227\ nm$. In contrast compound **1** dissolved in 10 mM phosphate buffer with 1 vol% DMSO at pH 7.4 shows positive CD bands at $\lambda = 295\ nm, 288\ nm$ and $234\ nm$ and negative bands at $\lambda = 283\ nm$ and $275\ nm$. These suggest different aggregates initially formed or present under the different sample preparation conditions. The CD spectra of the final state of both polymerisations however show identical signals (Figure S6 and S7) indicating the formation of the same end structure from different initial states.

For compound **1** dissolved in 10 mM phosphate buffer with 1 vol% HFIP at pH 7.4 an initial state could not be resolved on the timescale of the experiment. However, the CD signals of the polymerisation show only signals that coincide with the signals of the starting state of compound **1** lyophilised from DMSO and dissolved in 10 mM phosphate buffer, but do not convert to the signals of the final state of the other polymerisations observed. This observation suggests the stabilisation of the initial structure by hexafluoroisopropanol.

These findings support our hypothesis that the choice of solvent and solvent composition as well as sample preparation conditions have a considerable influence on the kinetic pathways of a supramolecular polymerization of peptide amphiphiles.

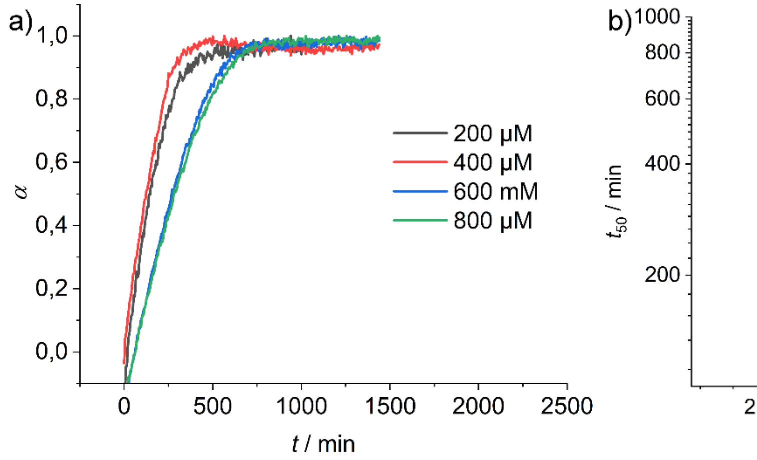

Figure S9. a) Degree of aggregation for the kinetic measurement of compound **1** in 10 mM phosphate buffer with 1 vol% DMSO content at pH 7.4 at 5 °C. b) double logarithmic plot of the half times t_{50} of the self-assembly of compound **1** at different monomer concentration c_m. Linear fit of the data points shows a slope of $m = 0.57$.

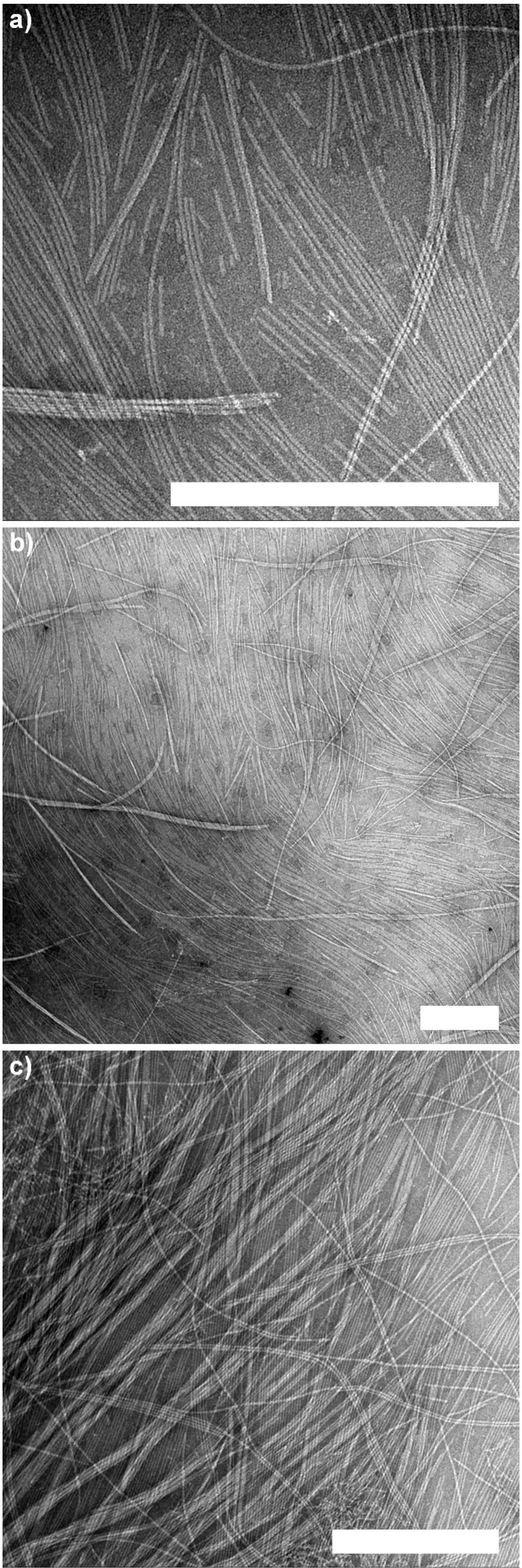

Figure S10. TEM micrographs of different magnification of a 400 μM solution of compound **1** in 10 mM phosphate buffer with 1 vol% DMSO content at pH 7.4; scale bar represents 500 nm. The absolute length and length distribution cannot be measured because the ends of the fibres cannot be resolved in TEM. a) small fibres are spaced out but the longer fibres have a higher tendency to laterally aggregate. b) long fibres bundle up to form thicker bundles. c) sample region that shows high number of bundled fibres. The overall length of the fibres is >1 μm.

2 Materials and General Methods

2.1 Solvents

All solvents were purchased in the best quality available and were used without further purification. Absolute DCM was purchased from *Acros Organics* (New Jersey, USA).

2.2 Degasification

For preparation of degassed solvents the freeze-pump-thaw-technique was used. In this method solvents were attached to a Schlenkline, frozen in liquid nitrogen and the gas atmosphere was pumped off. The stopcock was closed and the liquid nitrogen removed to let the liquid thaw and let the gas phase saturate with dissolved gas. The procedure was repeated at least three times.

2.3 Column chromatography

For column chromatography silica gel with a particle size of 0.035–0.070 mm and a pore size of 60 Å by *Acros Organics* (New Jersey, USA) was used. The eluents were freshly prepared of technical grade solvents, unless stated otherwise. For flash chromatography a pressure of 0.2–0.6 bar was applied with nitrogen. The analysis of the collected fractions was performed *via* thin layer chromatography.

2.4 Thin layer chromatography

Thin layer chromatography was performed on silica coated aluminum sheets *ALUGRAM Xtra SIL G/UV*$_{254}$ by *Macherey-Nagel* (Düren, Germany). The analytes were detected using UV-absorption or staining agents.

UV-absorption	λ = 254 nm.
Sugar-stain	solution of 0.25 mL *m*-methoxyphenol and 6.75 mL concentrated sulfuric acid in 250 mL ethanol.
$KMnO_4$-stain	solution of 6 g $KMnO_4$, 40 g K_2CO_3 and 13 mg NaOH in 600 mL water.
Hanessians stain	solution of 1 g $Ce(SO_4)_2$, 25 g $(NH_4)_6Mo_7O_{24}\cdot 4H_2O$ and 50 mL saturated sulfuric acid in 450 mL water.
Vanillin-stain	solution of 5 g vanillin, 60 mL acetic acid and 20 mL saturated sulfuric acid in 600 mL methanol.
Ninhydrin-stain	solution of 1.5 g ninhydrin and 15 mL acetic acid in 500 mL methanol.

Staining was achieved upon heating.

2.5 NMR spetroscopy

All ^{1}H- and ^{13}C-NMR spectra and corresponding 2D NMR-experiments were performed on an *Avance* II 400 (^{1}H-NMR: 400 MHz, ^{13}C-NMR 101 MHz) spectrometer or *Avance* III HD 400 (^{1}H-NMR: 400 MHz, ^{13}C-NMR 101 MHz) spectrometer by *Bruker* (Rheinstätten, Germany) equipped with a 5 mm broadband observe probehead (z gradient) or *Avance* III 600 (^{1}H-NMR: 600 MHz, ^{13}C-NMR 151 MHz) spectrometer by *Bruker* (Rheinstätten, Germany) equipped with a 5 mm cryogenic triple-band inverse probehead (z gradient) using standard Bruker release pulse sequences. All samples were dissolved in deuterated solvents by *Deutero* (Kastellaun, Germany). Evaluations of the spectra were carried out with *MestReNova* 14.1.0 by *Mestrelab Research S.L.* (Santiago de Compostela, Spain). The chemical shifts of the signals were locked relative to the residual solvent peaks reported in literature[1] and are given in parts per million (ppm) relative to tetramethylsilane (0 ppm). Coupling constants were measured in Hz and the nomenclature of the multiplicity of the signals was used as follows:

br = broad signal, s = singlet, d = doublet, t = triplet, q = quartet, quint = quintet and

m = multiplet.

2.6 Size exclusion chromatography

For size exclusion chromatography *Sephadex*™ LH-20 by *GE Healthcare* (Pittsburgh, USA) or *Bio-Beads*® S-X1 by *Bio Rad Laboratories GmbH* (Feldkirchen, Germany) was used. The

eluent used is stated individually. The analysis of the collected fraction was performed *via* thin layer chromatography.

2.7 Mass spectrometry

All mass spectra were recorded on an G6545A Q-ToF-MS by *Agilent* (Santa Clara, USA), ionized using electronspray ionization (ESI). Sample injection is performed via 1260 Infinity II HPLC-System by Agilent (Santa Clara, USA) with G7111B 1260 Quarternary Pump, G7129A 1260 Vialsampler and G7116A 1260 Multicolumn Thermostat.

2.8 High performance liquid chromatography (HPLC)

The HPLC consists of a PU-4086 semi-preparative binary pump module, an AS-4050 HPLC Autosampler, a MD-4015 Photo Diode Array Detector, a CO-4060 column oven by *Jasco* (Pfungstadt, Germany) and a CHF122SC fraction collector by *Advantec MFS Inc.* (Dublin, USA).

Method **A**

Analytical HPLC using NUCLEODUR C18 Pyramid column by *Macherey Nagel* was performed at a flowrate of 1 mL/min with the solvents and the time program **A**, shown below. The particle size of the column is 1.8 µm, the length 50 mm and the diameter 4.6 mm.

Table S1. Gradient of the solvents used in Method **A**.

Time /min	1mM NH_4HCO_3 in ultrapure water /vol%	95% Acetonitrile 5% ultrapure water /vol%
0	80	20
0.65	80	20
5.65	20	80
15	20	80

Method **B**

Purification using NUCLEODUR C18 Pyramid by *Macherey-Nagel* was performed at a flowrate of 18.9 mL/min with the solvents and the time program **B**, shown below. The particle size of the column is 5 µm, the length 250 mm and the diameter 21 mm.

Table S2. Gradient of the solvents used in Method **B**.

Time /min	1mM NH_4HCO_3 in ultrapure water /vol%	95% Acetonitrile 5% ultrapure water /vol%

0	80	20
8	80	20
32	20	80
40	20	80

Method **C**

Purification using NUCLEODUR C18 Pyramid by *Macherey-Nagel* was performed at a flowrate of 18.9 mL/min with the solvents and the time program **C**, shown below. The particle size of the column is 5 µm, the length 250 mm and the diameter 21 mm.

Table S3. Gradient of the solvents used in Method **C**

Time /min	ultrapure water + 0.1% TFA /vol%	Acetonitrile + 0.1%TFA /vol%
0	100	0
8	100	0
32	0	100
40	0	100

2.9 Solid phase peptide synthesis

Solid phase peptide synthesis was carried out on a CS136XT Peptide Synthesizer by CS Bio Co. (Menlo Park, USA). Peptide grade solvents and reagents were used. Peptide synthesis was carried out by a modified version of the standard Fmoc-protocol shown by *Atherton* and *Sheppard*.[2]

2.10 Centrifugation

Centrifugation was performed on a VWR *MicroStar* 12 by *Avantor* (Darmstadt, Germany) using 1.5 mL Eppendorf-tubes, or on a *VWR Mega Star* 600 by *Avantor* (Darmstadt, Germany) using 15 mL or 50 mL Falcon-tubes.

2.11 Circular Dichroism (CD) Spectroscopy

Circular dichroism spectroscopy was performed on a J-815 CD-Spectrometer by *Jasco* (Pfungstadt, Germany) using a 1 mm, 2 mm or 10 mm glass cuvette. Spectra were processed with *Origin 2022* by *OriginLab* (Northampton, USA). Spectra were measured at 5 °C in the range of 190 nm to 350 nm.

2.12 Compound 1 sample preparation

Compound **1** containing fractions were collected after HPLC purification as described in Chapter 3 and CH_3CN was removed under reduced pressure. H_2O and NH_4HCO_3 were removed *via* lyophilisation to yield a colorless lyophilisate. A 1000 µM stock solution of compound **1** was prepared in CH_3CN:H_2O (1:1, *v*:*v*) and used for serial dilution to yield samples of the respective concentration. The samples were lyophilized and dissolved in the respective solvent (mixture) or buffer used for the individual experiments. All samples were prepared from the same stock solution and do not differ to this point. Additional sample preparation is stated with the respective experiment.

2.13 Kinetic CD spectroscopy experiments

All samples were used as lyophilisates prepared according to ESI 2.12 and dissolved in DMSO to measure reference CD-spectra of disassembled species. DMSO solution were lyophilized again to yield dry colorless powders. Before each measurement the sample and the solvent were pre-tempered in an ice bath at 5 °C and the cuvette in the sample holder of the instrument. For the kinetic measurement the sample was dissolved in 10 mM phosphate buffer (pH 7.4) and measured immediately. Full CD-spectra were recorded every 5 min at a scanning speed of 50 nm/min. The spectra were corrected by substraction of the background (solvent and cuvette). The degree of aggregation α was calculated using formula (1).

$$\alpha(t) = \frac{\Delta\epsilon^{295}(t) - \Delta\epsilon^{295}_{DMSO}}{\Delta\epsilon^{295}_{min} - \Delta\epsilon^{295}_{DMSO}} \qquad (1)$$

Where $\Delta\epsilon^{295}(t)$ is the measured CD signal at the wavelength $\lambda = 295\ nm$ with time t, $\Delta\epsilon^{295}_{min}$ is the minimal CD signal of all time points at $\lambda = 295\ nm$ and $\Delta\epsilon^{295}_{DMSO}$ is the CD signal of the reference sample of each concentration in DMSO at $\lambda = 295\ nm$. The half time of the self-assembly t_{50} can be found when fitting the resulting plots with a logistic fit with formula (2).

$$y = \frac{A_1 - A_2}{1 + \left(\frac{t}{t_{50}}\right)^p} + A_2 \qquad (2)$$

Where A_1 is the upper asymptote, A_2 is the lower asymptote and p is the power.

2.14 pH Titration CD measurements

For the pH Titration a 400 µM solution of compound **1** in MeCN:H_2O (1:1) was used. The starting pH of the solution was 6.18. The pH was adjusted to pH 12.17 using aqueous 1 M NaOH. Starting from basic pH the solution was adjusted stepwise to pH 3.18 using aqueous 0.1 M HCl. CD spectra were measured at each integer pH value. All spectra were

averaged over three replicates and corrected by substraction of the background (solvent and cuvette).

2.15 Length distribution

The length of individual fibres was measured using *ImageJ 1.53t* (Wayne Rasband, National Institutes of Health, USA). The number average fibre length L_n, the weighted average fibre length L_w and the Dispersity *Đ* were calculated using formula (3).

$$L_n = \frac{\sum_{i=1}^{n} n_i L_i}{\sum_{i=1}^{n} n_i} \qquad L_w = \frac{\sum_{i=1}^{n} n_i {L_i}^2}{\sum_{i=1}^{n} n_i L_i} \qquad Đ = \frac{L_w}{L_n} \tag{3}$$

Where n is the number of fibres measured and L the measured length in nm.

3 Synthesis

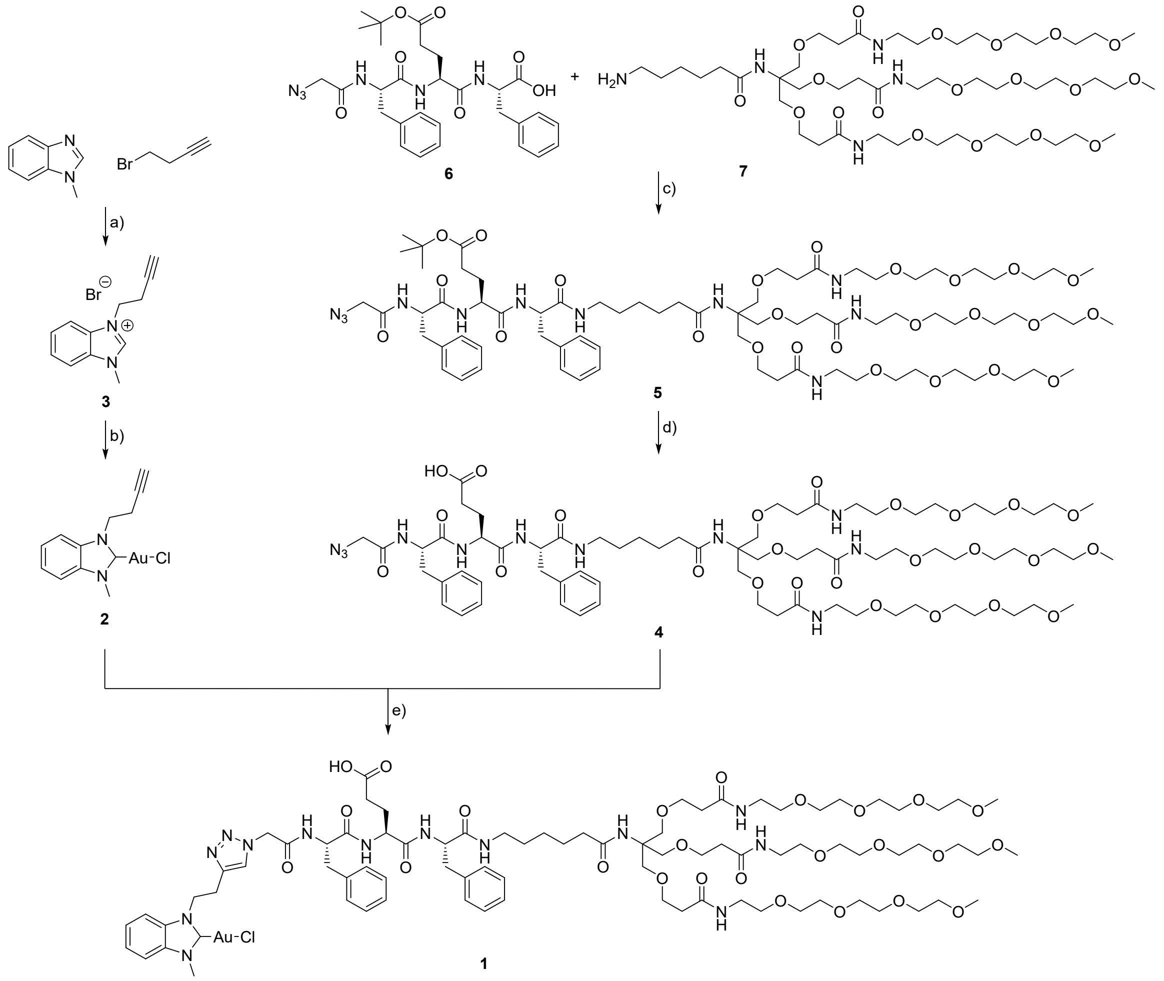

Figure S11: Synthetic route for the synthesis of compound **1**. a) 1-Methyl-1*H*-benzo[*d*]-imidazole (1.0 eq.), 4-bromobut-1-yne (1.5 eq.), toluene, 110 °C, 72 h; b) 1) **3** (1.0 eq.), Ag_2O (0.6 eq.), DCM, r.t., 68 h, 2) Au(I)Cl(tht) (1.0 eq.), NaCl (1.5 eq.), r.t., 2.5 h; c) **6** (1.3 eq.), **7** (1.0 eq.), PyBOP (1.5 eq.), HOBt · H_2O (1.5 eq.), DMF, 0 °C – r.t., over night; d) **5** (1.0 eq.), TFA:H_2O, 1:1, r.t., 2 h; e) **2** (1.1 eq.), **4** (1.0 eq.), $CuSO_4$ · $5H_2O$ (0.2 eq.), TBTA (0.2 eq.), sodium L-ascorbate (0.2 eq.), THF:H_2O, 1:1, 45 °C, 7 h.

Compound **6** and compound **7** were synthesized as reported earlier.[3][4]

Compound 1

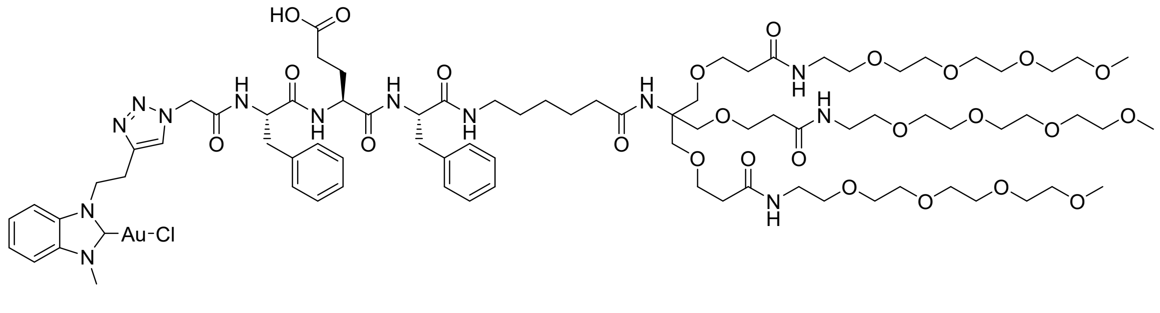

The reaction was performed under SCHLENK conditions with degassed solvents under exclusion of light. Compound **4** (140.0 mg, 0.092 mmol, 1.0 eq.) and compound **2** (42.1 mg, 0.101 mmol, 1.1 eq.) were dissolved in THF:H_2O, 1:1 (vol%, 4 mL). $CuSO_4 \cdot 5H_2O$ (4.6 mg, 0.018 mmol, 0.2 eq.), TBTA (9.7 mg, 0.018 mmol, 0.2 eq.) and sodium L-ascorbate (3.7 mg, 0.018 mmol, 0.2 eq.) were dissolved in THF:H_2O, 1:1 (vol%, 5 mL) separately and added to the solution. The mixture was stirred at 45 °C for 7 h until full conversion (followed by HPLC (Method **A**)). The reaction mixture was concentrated under reduced pressure and lyophilized. The crude product was dissolved in DMSO, centrifuged to remove gold nanoparticles and purified via HPLC (Method **B**) to yield a colorless lyophilisate.

Yield: 82.8 mg (0.043 mmol, 47 %) colorless lyophilisate.

MF $C_{83}H_{129}AuClN_{13}O_{25}$ MW 1941.43 [1939.86].

HR-TOF-MS (ESI, pos.), *m*/*z*: 1927.8720 $[M\text{-}H\text{-}Cl\text{+}Na]^+$ calc. 1927.8780.

HR-TOF-MS (ESI, neg.), *m*/*z*: 1903.8663 $[M\text{-}Cl\text{-}2H]^-$ calc. 1903.8815.

^{1}H NMR (600 MHz, DMSO-d_6, 296 K, COSY) δ/ppm:12.15 (bs, 1H, COO*H*), 8.32 (bs, 1H, N*H*^{Glu}), 8.01 (bs, 1H, N*H*^{Phe}), 7.99–7.89 (m, 5H, N*H*^{Phe}, 3xN*H*^{Dnd}, N*H*^{Ahx}), 7.85 (d, 1H, *J* = 7.6 Hz, *H*-7^{BzIm}), 7.67(d, 1H, *J* = 8.0 Hz, *H*-4^{BzIm}), 7.54–7.44(m, 2H, *H*-5^{BzIm}, *H*-6^{BzIm}), 7.24–7.16 (m, 4H, 2xPhe$^{\delta}$, 2xPhe$^{\varepsilon}$), 7.16–7.08 (m, 3H, 2xPhe$^{\varepsilon}$, Phe$^{\zeta}$), 7.07–7.02 (m, 2H, Phe$^{\delta}$), 6.92 (t, 1H, *J* = 7.4 Hz, Phe$^{\zeta}$), 5.00–4.80(m, 4H, Gly$^{\alpha}$, N^{BzIm}C*H*$_2$), 4.56 (q, 1H, *J* = 7.6 Hz, Phe$^{\alpha}$), 4.45(q, 1H, *J* = 7.6 Hz, Phe$^{\alpha}$), 4.22 (q, 1H, *J* = 7.4 Hz, Glu$^{\alpha}$), 3.76 (s, 3H, N^{BzIm}C*H*$_3$), 3.56–3.51 (m, 12H, 3xC$_q$C*H*$_2$^{Tris},3xCOCH$_2$C*H*$_2$^{Dnd}), 3.50–3.48(m, 30H, 15xOC*H*$_2$^{Dnd}), 3.42–3.37(m, 12H, 6xOC*H*$_2$^{Dnd}), 3.34–3.28 (m, 2H, N^{BzIm}CH$_2$C*H*$_2$), 3.22 (s, 9H, OC*H*$_3$^{Dnd}), 3.19(q, 6H, *J* = 5.9 Hz, NHC*H*$_2$^{Dnd}), 3.08–3.01(m, 1H, Ahx$^{\varepsilon'}$), 2.95–2.86(m, 3H, Ahx$^{\varepsilon''}$,2xPhe$^{\beta'}$), 2.80 (dd, 1H, *J* = 13.6 Hz, *J* = 8.1 Hz, Phe$^{\beta''}$), 2.71 (q, 1H, *J* = 13.4 Hz, *J* = 9.2 Hz, Phe$^{\beta''}$), 2.29 (t, 6H, *J* = 6.5 Hz, 3xCOC*H*$_2$^{Dnd}), 2.16 (t, 2H, *J* = 7.1 Hz, Glu$^{\gamma}$), 2.03 (t, 2H, *J* = 7.5 Hz, Ahx$^{\alpha}$), 1.86–1.78 (m, 1H, Glu$^{\beta'}$), 1.75–1.65 (m, 1H, Glu$^{\beta''}$), 1.39(p, 2H, *J* = 7.4 Hz, Ahx$^{\beta}$), 1.29 (p, 2H, *J* = 6.8 Hz, Ahx$^{\delta}$), 1.13 (p, 2H, *J* = 6.8 Hz, Ahx$^{\gamma}$).

^{13}C NMR (151 MHz, DMSO-d_6, 296 K, HSQC, HMBC) δ/ppm: 195.04 (*C*Au), 174.03 (*C*OO^{Glu}), 172.45 (*C*=O^{Tris}), 170.86 (*C*=O^{Phe}), 170.66 (*C*=O^{Glu}), 170.36 (*C*=O^{Phe}), 170.30 (*C*=O^{Ahx}), 166.85 (*C*=O^{Gly}), 137.54, 138.39 (Phe$^{\gamma}$), 133.72, 132.70 (*C*-3a^{BzIm}, *C*-7a^{BzIm}), 129.22, 129.19 (Phe$^{\varepsilon}$), 128.07, 127.91 (Phe$^{\delta}$), 126.31, 126.09 (Phe$^{\zeta}$), 124.23, 124.06 (*C*-5^{BzIm}, *C*-6^{BzIm}), 111.92 (*C*-4^{BzIm}, *C*-7^{BzIm}), 71.31, 69,84, 69.82, 69.76, 69.63, 69.17 (OCH$_2$^{EG}), 68.28 (OCH$_2$^{Tris}),

67.35 ($COCH_2CH_2^{Dnd}$), 59.54 (C_q^{Tris}), 58.09 (OCH_3^{Dnd}), 53.96 (Glu^{α}), 53.84 (Gly^{α}), 53.64 (Phe^{α}), 52.03 (Phe^{α}), 48.57 ($N^{BzIm}CH_2$), 38.56 ($NHCH_2^{Dnd}$), 38.01 (Ahx^{ε}), 37.79 (Phe^{β}), 35.86 (Ahx^{α}, $COCH_2^{Dnd}$), 34.39 ($N^{BzIm}CH_3$), 30.22 (Glu^{γ}), 28.82 (Ahx^{δ}), 27.51 (Glu^{β}), 26.06 (Ahx^{γ}), 25.06 (Ahx^{β}).

Compound 2

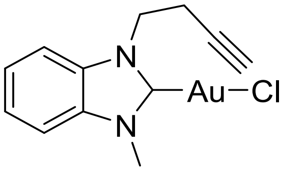

The reaction was performed under SCHLENK conditions with degassed solvents under exclusion of light. Compound **3** (200.0 mg, 0.754 mmol, 1.0 eq.) and Ag_2O (104.9 mg, 0.453 mmol, 0.6 eq.) were mixed in dry DCM (9 mL) and stirred for 68 h at room temperature. Au(I)Cl(tht) (241.8 mg, 0.754 mmol, 1.0 eq.) and NaCl (66.1 mg, 1.131 mmol, 1.5 eq.) were added with argon counterflow and stirred for another 2.5 h. The the suspension was filtered and washed with DCM. The filtrate was concentrated and precipitated in diethyl ether at -20 °C. the precipitate was centrifuged for 10 min at 13500 rpm and the supernatant was discarded to yield an off-white crystalline solid.

Yield: 184 mg (0.441 mmol, 58 %) off-white crystalls.

MF $C_{12}H_{12}AuClN_2$ MW 416.66 [416.0355].

HR-TOF-MS (ESI, pos.), *m*/*z*: 422.0926 $[M\text{-}Cl\text{+}ACN]^+$ calc. 422.0931,

381.0665 $[M\text{-}Cl]^+$ calc. 381.0666.

^1H NMR (300 MHz, $CDCl_3$, 296 K, COSY) δ/ppm: 7.61–7.55 (m, 1H, $H\text{-}4^{BzIm}$), 7.51–7.42 (m, 3H, $H\text{-}5^{BzIm}$, $H\text{-}6^{BzIm}$, $H\text{-}7^{BzIm}$), 4.63 (t, 2H, J = 6.9 Hz, $N^{BzIm}CH_2$), 4.06 (s, 3H, $N^{BzIm}CH_3$), 2.88 (td, 2H, J = 6.9 Hz, J = 2.6 Hz, $N^{BzIm}CH_2CH_2$), 2.00 (t, 1H, J = 2.7 Hz, CH^{alkyne}),

^{13}C NMR (75 MHz, $CDCl_3$, 296 K, HSQC, HMBC) δ/ppm: 178.9 (*C*Au), 133.8, 133.2 ($C\text{-}3a^{BzIm}$, $C\text{-}7a^{BzIm}$), 124.9, 124.8 ($C\text{-}5^{BzIm}$, $C\text{-}6^{BzIm}$), 111.7 ($C\text{-}4^{BzIm}$), 111.5 ($C\text{-}7^{BzIm}$), 79.7 (C_q^{alkyne}), 72.3 (CH^{alkyne}), 47.0 ($N^{BzIm}CH_2$), 35.5 ($N^{BzIm}CH_3$), 20.6 ($N^{BzIm}CH_2CH_2$).

Compound 3

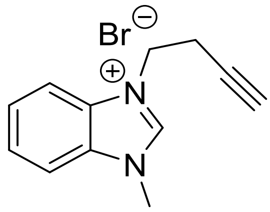

1-Methyl-1*H*-benzo[*d*]-imidazole (356.5 mg, 2.699 mmol, 1.0 eq.) was dissolved in 10 mL toluene. 4-bromobut-1-yne (538.5 mg, 4.049 mmol, 1.5 eq.) was added and the reaction mixture was stirred at 110 °C for 72 h. The precipitated salt was filtered and washed with diethyl ether (3x 5 mL). The product was dried under reduced pressure to yield an off-white amorphous solid.

Yield: 454 mg (1.719 mmol, 64 %) off-white amorphous solid.

MF $C_{12}H_{13}BrN_2$ MW 265.15 [264.0262].

HR-TOF-MS (ESI, pos.), *m*/*z*: 185.1071 $[M\text{-}Br]^+$ calc. 185.1073.

^1H NMR (400 MHz, $CDCl_3$, 296 K, COSY) δ/ppm: 11.23 (s, 1H, H-2^{BzIm}), 7.87–7.80 (m, 1H, H-4^{BzIm}), 7.77–7.71 (m, 1H, H-7^{BzIm}), 7.68–7.61 (m, 2H, H-5^{BzIm}, H-6^{BzIm}), 4.84 (t, 2H, J = 6.5 Hz, $N^{BzIm}CH_2$), 4.29 (s, 3H, $N^{BzIm}CH_3$), 3.02 (td, 2H, J = 6.5 Hz, J = 2.7 Hz, $N^{BzIm}CH_2CH_2$), 2.05 (t, 1H, J = 2.6 Hz, CH^{alkyne}).

^{13}C NMR (101 MHz, $CDCl_3$, 296 K, HSQC, HMBC) δ/ppm: 143.4 (C-2^{BzIm}), 131.9, 131.4 (C-$3a^{BzIm}$, C-$7a^{BzIm}$), 127.4, 127.4 (C-5^{BzIm}, C-6^{BzIm}), 113.4 (C-4^{BzIm}), 113.0 (C-7^{BzIm}), 78.9 (C_q^{alkyne}), 73.1 (CH^{alkyne}), 46.1 ($N^{BzIm}CH_2$), 34.1 ($N^{BzIm}CH_3$), 20.4 ($N^{BzIm}CH_2CH_2$).

Compound 4

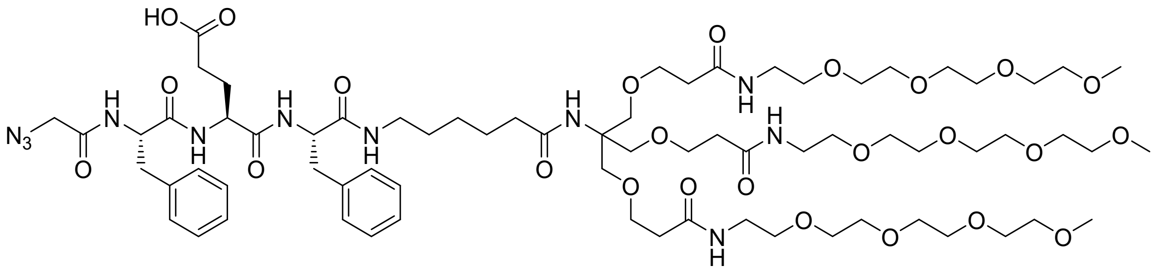

Compound **5** (199 mg, 0.126 mmol, 1.0 eq.) was dissolved in TFA:H_2O, 1:1 (vol%, 10 mL) and stirred at room temperature for 2 h. Solvents were removed *in vacuo* and the crude product was co-distilled with toluene (3x12 mL) and dried under reduced pressure. The product was purified *via* HPLC (Method **C**) to yield a colorless lyophilisate.

Yield: 100 mg (0.066 mmol, 52 %) colorless lyophilisate.

MF $C_{71}H_{117}N_{11}O_{25}$ MW 1524,77 [1523,8222].

HR-TOF-MS (ESI, pos.), *m*/*z*: 1546.8114 $[M+Na]^+$ calc. 1546.8114,

784.9010 $[M+2Na]^{2+}$ calc. 784.9003.

^1H NMR (400 MHz, DMSO-d_6, 296 K, COSY) δ/ppm: 8.32 (d, 1H, J = 8.3 Hz, NH^{Phe}), 8.26 (d, J = 7.8 Hz, 1H, NH^{Glu}), 7.95–7.87 (m, 5H, 3xNH^{Dnd}, NH^{Phe}, NH^{Ahx}), 7.24–7.13 (m, 10H, 4xPhe^{δ}, 4xPhe^{ε}, 2xPhe^{ζ}), 6.98 (s, 1H, NH^{Tris}), 4.60–4.53 (m, 1H, Phe^{α}), 4.45 (q, 1H, J = 7.7 Hz, Phe^{α}), 4.23 (q, 1H, J = 7.5 Hz, Glu^{α}), 3.82–3.69 (m, 2H, Gly^{α}), 3.58–3.46 (m, 36H, 18xOCH_2^{EG}), 3.43–3.37 (m, 6H, 3x$NHCH_2CH_2^{Dnd}$), 3.23 (s, 9H, 3xOCH_3^{Dnd}), 3.19 (q, 6H,

J = 5.8 Hz, NHCH_2^{Dnd}), 3.09–2.86 (m, 4H, Ahx^{ε}, 2xPhe$^{β'}$), 2.82 (dd, 1H, J = 13.6 Hz, J = 8.5 Hz, Phe$^{β''}$), 2.72 (dd, 1H, J = 14.0 Hz, J = 10.0 Hz, Phe$^{β''}$), 2.29 (t, 6H, J = 6.4 Hz, COCH_2^{Dnd}), 2.21–2.15 (m, 2H, Glu^{γ}), 2.04 (t, 2H, J = 7.5 Hz, Ahx^{α}), 1.90–1.79 (m, 1H, Glu$^{β'}$), 1.78–1.67 (m, 1H, Glu$^{β''}$), 1.40 (p, 2H, J = 7.6 Hz, Ahx^{β}), 1.29 (p, 2H, J = 7.4 Hz, Ahx^{δ}), 1.19–1.09 (m, 2H, Ahx^{γ}).

^{13}C NMR (101 MHz, DMSO-d_6, 296 K, HSQC, HMBC) $δ$/ppm: 174.0 (COOH), 172.4 (C=O^{Phe}), 170.9 (C=O^{Phe}), 170.6 (C=O^{Glu}), 170.3 (C=O^{Ahx}), 170.3 (C=O^{Dnd}), 167.3 (C=O^{Gly}), 137.6, 137.6 (Phe^{γ}), 129.2 (Phe^{ε}), 128.1 (Phe^{δ}), 126.3 (Phe^{ζ}), 71.3, 69.8, 69.7, 69.6, 69.1 (OCH$_2$^{EG}), 68.3 (OCH$_2$^{Tris}), 67.3 (COCH$_2$CH$_2$^{Dnd}), 59.5 (C_q^{Tris}), 58.1 (OCH$_3$^{EG}), 53.9, 53.8 (Phe^{α}), 52.1 (Glu^{α}), 50.5 (Gly^{α}), 38.5 (Ahx^{ε}, NHCH$_2$^{Dnd}), 37.9, 37.5 (Phe^{β}), 35.9 (Ahx^{α}, COCH$_2$^{Dnd}), 30.1 (Glu^{γ}), 28.8 (Ahx^{δ}), 27.4 (Glu^{β}), 26.0 (Ahx^{γ}), 25.0 (Ahx^{β}).

Compound 5

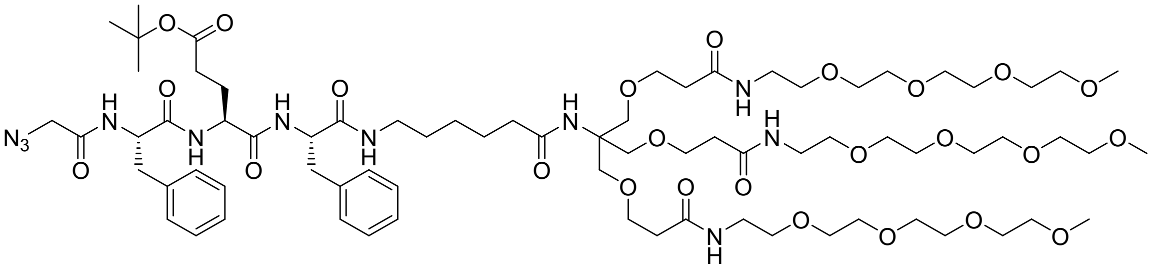

Compound **6** (165.1 mg, 0.223 mmol, 1.3 eq.) and compound **7** (175.0 mg, 0.172 mmol, 1.0 eq.) were dissolved in peptide grade DMF (1 mL) and PyBOP (134.2 mg, 0.258 mmol, 1.5 eq.) and HOBt · H_2O (34.8 mg, 0.258 mmol, 1.5 eq.) were added. The solution was cooled in an ice bath and DIPEA (44.9 µL, 0.258 mmol, 1.5 eq.) was added portionwise. The ice bath was removed and the reaction mixture was stirred at room temperature overnight. The solvent was removed *in vacuo* and the crude product was subjected to size exclusion chromatography (Sephadex LH-20, MeOH). The product was dissolved in H_2O and lyophilized to yield a colorless lyophilisate.

Yield: 215.0 mg (0.136 mmol, 79 %) colorless lyophilisate.

MF $C_{75}H_{125}N_{11}O_{25}$ MW 1580.88 [1579.8848].

HR-TOF-MS (ESI, pos.), m/z: 1602.8742 [M+Na]$^+$ calc. 1602.8740,

812.9328 [M+2Na]$^{2+}$ calc. 812.9316.

^{1}H NMR (400 MHz, DMSO-d_6, 296 K, COSY) $δ$/ppm: 8.32 (d, 1H, J = 8.2 Hz, NH^{Phe}), 8.26 (d, J = 7.9 Hz 1H, NH^{Glu}), 7.94–7.87 (m, 5H, 3xNH^{Dnd}, NH^{Phe}, NH^{Ahx}), 7.26–7.12 (m, 10H, 4xPhe^{δ}, 4xPhe^{ε}, 2xPhe^{ζ}), 6.98 (s, 1H, NH^{Tris}), 4.60–4.53 (m, 1H, Phe^{α}), 4.48–4.41 (m, 1H, Phe^{α}), 4.23 (q, 1H, J = 7.8 Hz, Glu^{α}), 3.80–3.69 (m, 2H, Gly^{α}), 3.59–3.45 (m, 36H, 18xOCH_2^{EG}), 3.43–3.38 (m, 6H, 3xNHCH$_2$CH_2^{Dnd}), 3.23 (s, 9H, 3xOCH_3^{Dnd}), 3.19 (q, 6H, J = 5.9 Hz, NHCH_2^{Dnd}), 3.10–2.89 (m, 4H, Ahx^{ε}, 2xPhe$^{β'}$), 2.81 (dd, 1H, J = 13.7 Hz, J = 8.4 Hz, Phe$^{β''}$), 2.72 (dd, 1H, J = 14.0 Hz, J = 10.0 Hz, Phe$^{β''}$), 2.29 (t, 6H, J = 6.4 Hz,

COCH_2^{Dnd}), 2.18–2.11 (m, 2H, Glu$^{\gamma}$), 2.04 (t, 2H, J = 7.5 Hz, Ahx$^{\alpha}$), 1.88–1.77 (m, 1H, Glu$^{\beta'}$), 1.77–1.64 (m, 1H, Glu$^{\beta''}$), 1.44–1.36 (m, 11H, 3xCH_3^{tBu}Ahx$^{\beta}$), 1.31 (p, 2H, J = 7.4 Hz, Ahx$^{\delta}$), 1.15 (p, 2H, J = 7.1 Hz, Ahx$^{\gamma}$).

^{13}C NMR (101 MHz, DMSO-d_6, 296 K, HSQC, HMBC) δ/ppm: 172.4 (C=O^{Phe}), 171.7 (C=O^{Phe}), 170.9 (C=O^{Glu}), 170.5 (C=O^{Ahx}), 170.3 (C=O^{Dnd}), 167.3 (C=O^{Gly}), 137.6, 137.6 (Phe$^{\gamma}$), 129.2 (Phe$^{\varepsilon}$), 128.1, 128.1 (Phe$^{\delta}$), 126.3, 126.3 (Phe$^{\zeta}$), 79.7 (C_q^{tBu}) 71.3, 69.8, 69.8, 69.7, 69.6, 69.1 (OCH_2^{EG}), 68.3 (OCH_2^{Tris}), 67.3 (COCH$_2$CH$_2^{Dnd}$), 59.5 (C_q^{Tris}), 58.1 (OCH_3^{EG}), 53.9, 53.8 (Phe$^{\alpha}$), 52.0 (Glu$^{\alpha}$), 50.5 (Gly$^{\alpha}$), 38.5 (Ahx$^{\varepsilon}$, NHCH$_2^{Dnd}$), 37.9, 37.5 (Phe$^{\beta}$), 35.9 (Ahx$^{\alpha}$, COCH$_2^{Dnd}$), 31.2 (Glu$^{\gamma}$), 28.8 (Ahx$^{\delta}$), 27.8, (CH_3^{tBu}), 27.4 (Glu$^{\beta}$), 26.0 (Ahx$^{\gamma}$), 25.0 (Ahx$^{\beta}$).

4 NMR spectra

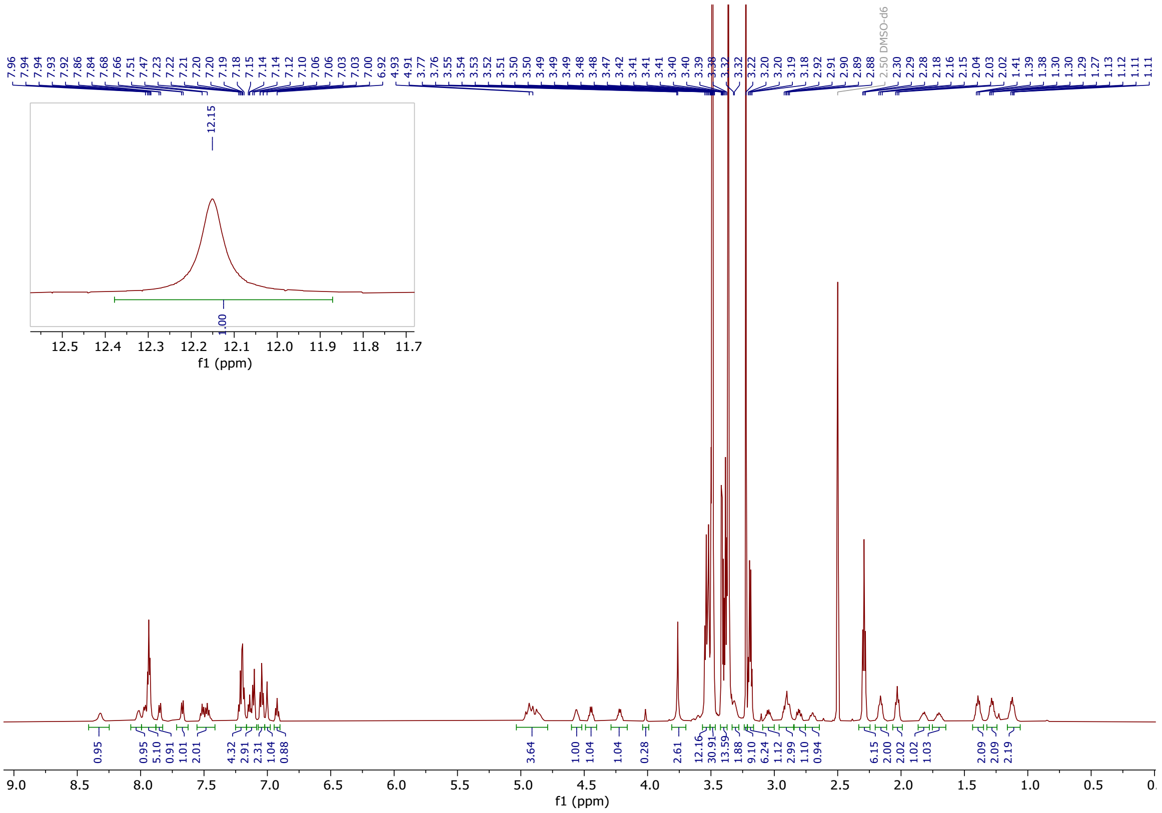

Figure S12: ^{1}H NMR spectrum of compound **1** in DMSO-d_6.

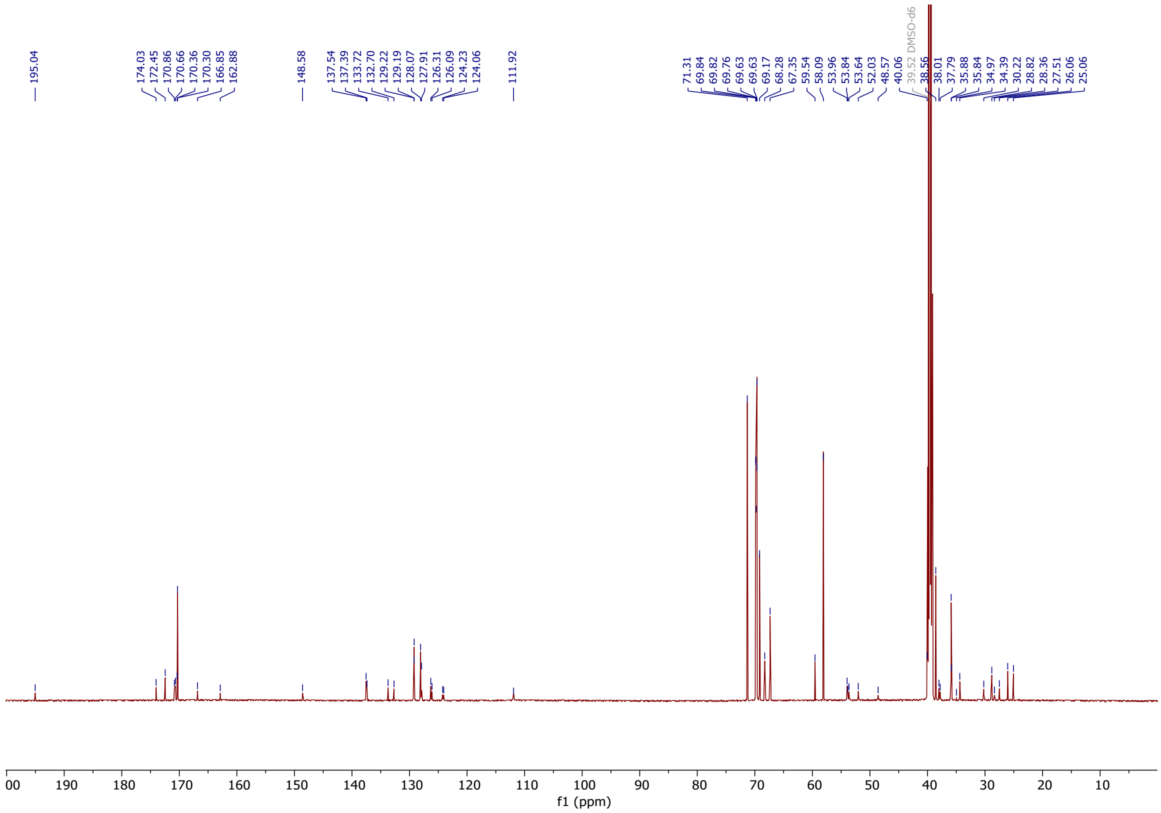

Figure S13: ^{13}C NMR of compound **1** in DMSO-d_6.

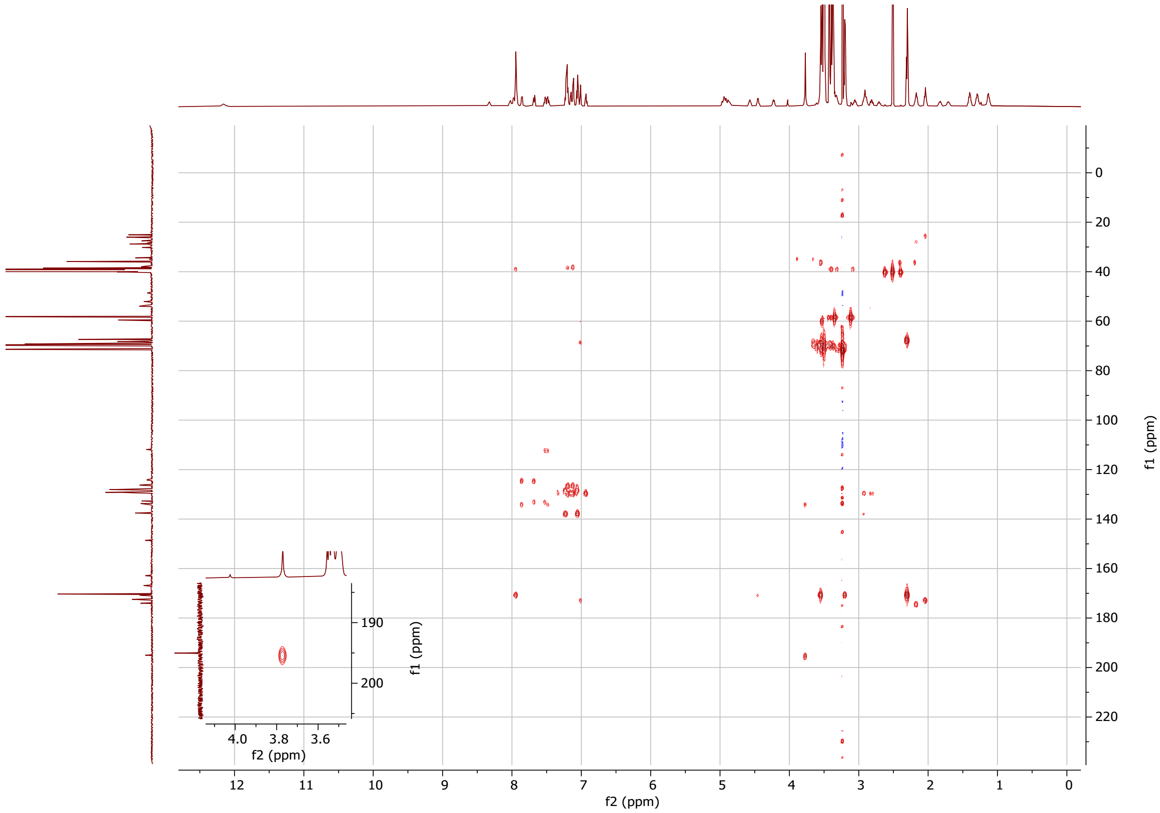

Figure S14: 2D-HMBC NMR spectrum of compound **1** in DMSO-d_6.

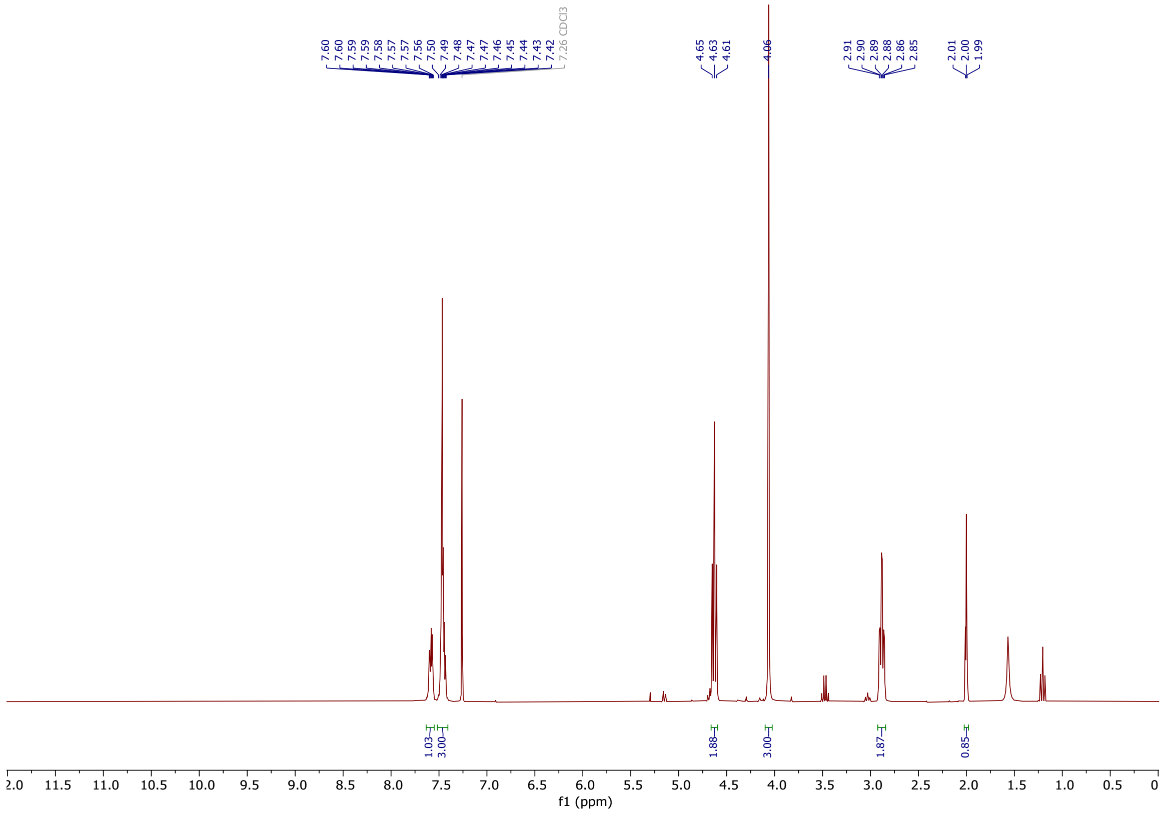

Figure S15: ^{1}H NMR spectrum of compound **2** in $CDCl_3$.

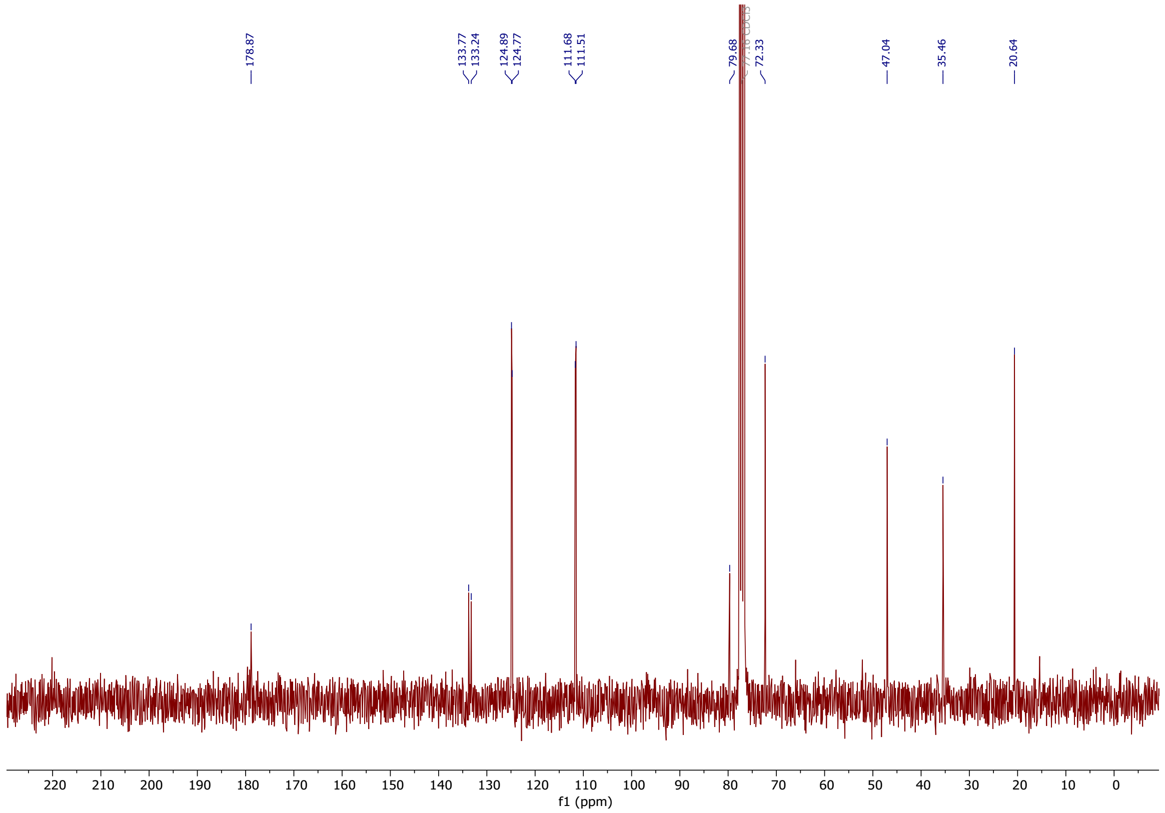

Figure S16: ^{13}C spectrum of compound **2** in $CDCl_3$.

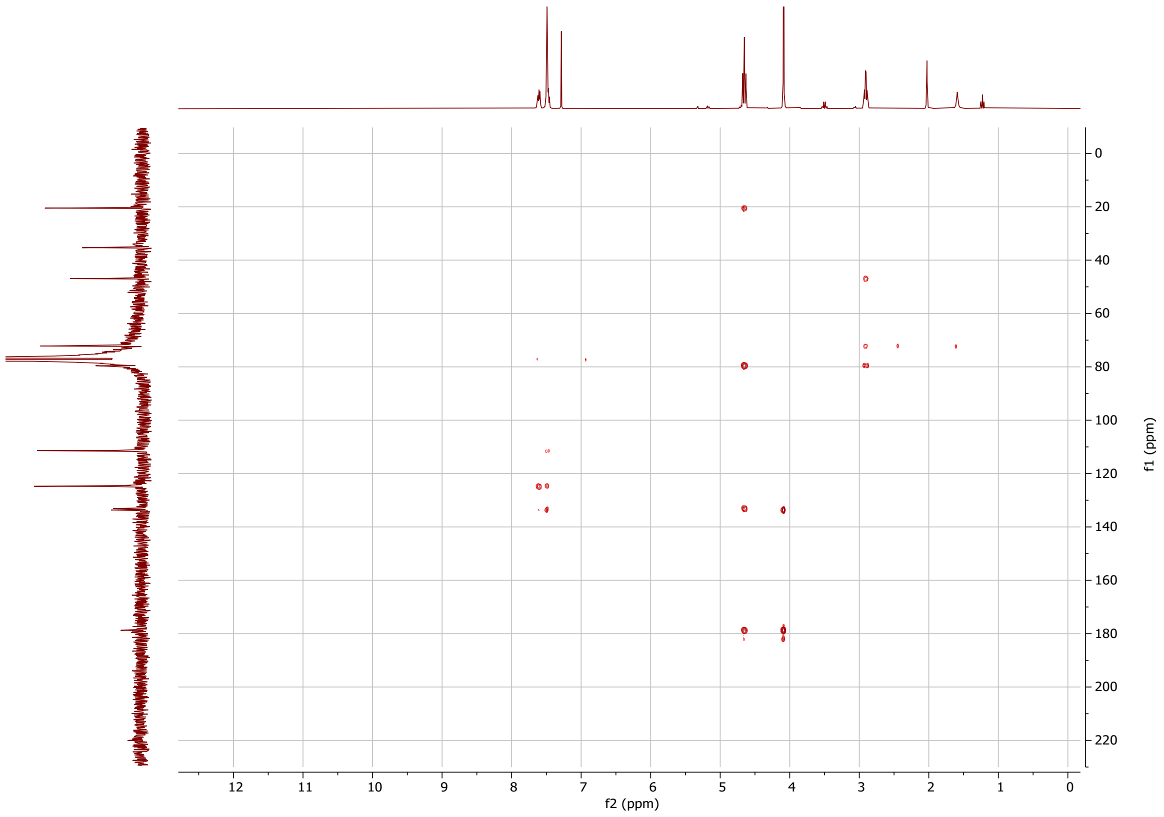

Figure S17: 2D-HMBC NMR spectrum of compound **2** in $CDCl_3$.

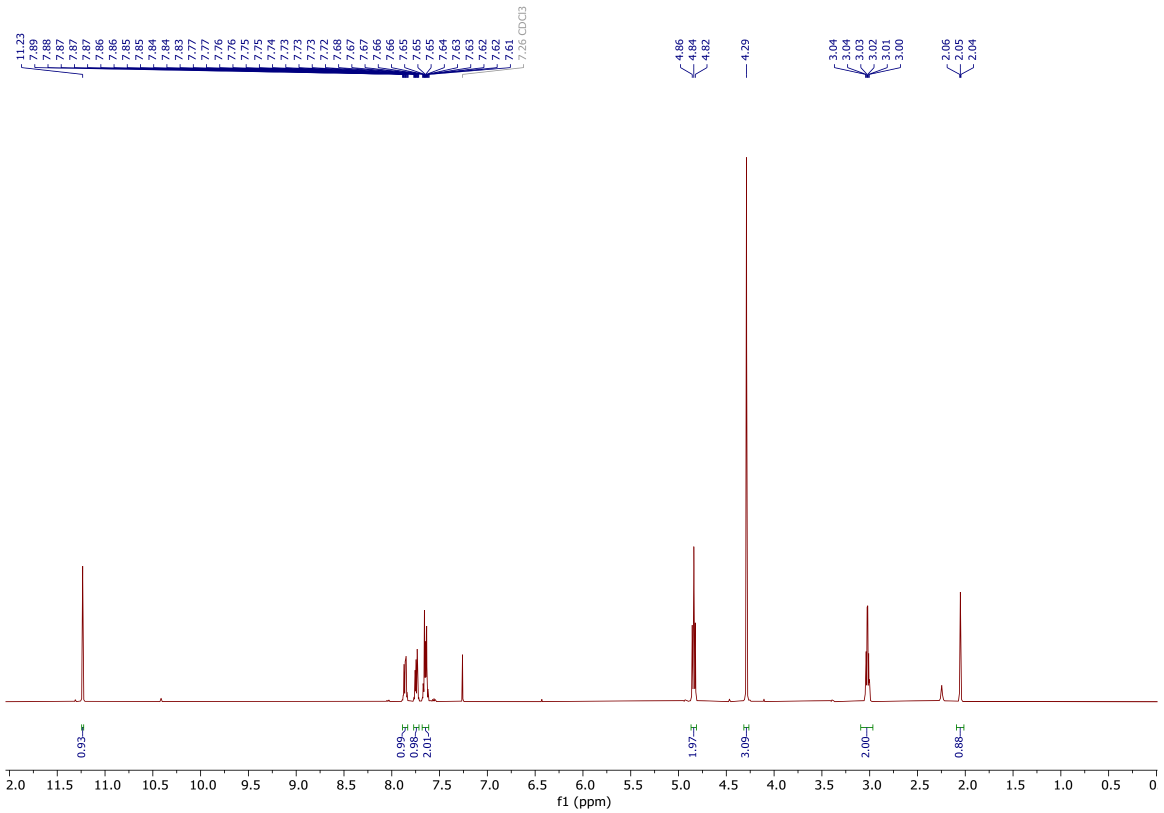

Figure S18: ^1H NMR spectrum of compound **3** in $CDCl_3$.

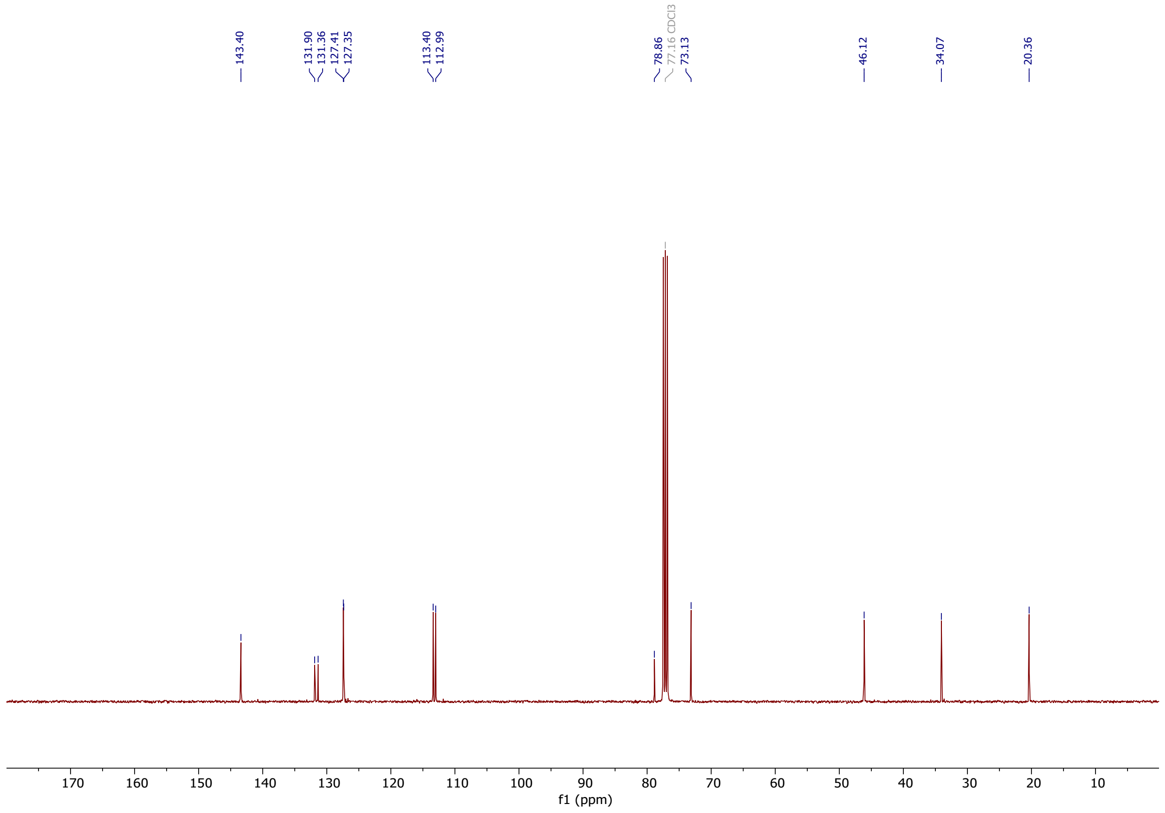

Figure S19: ^{13}C NMR spectrum of compound **3** in $CDCl_3$.

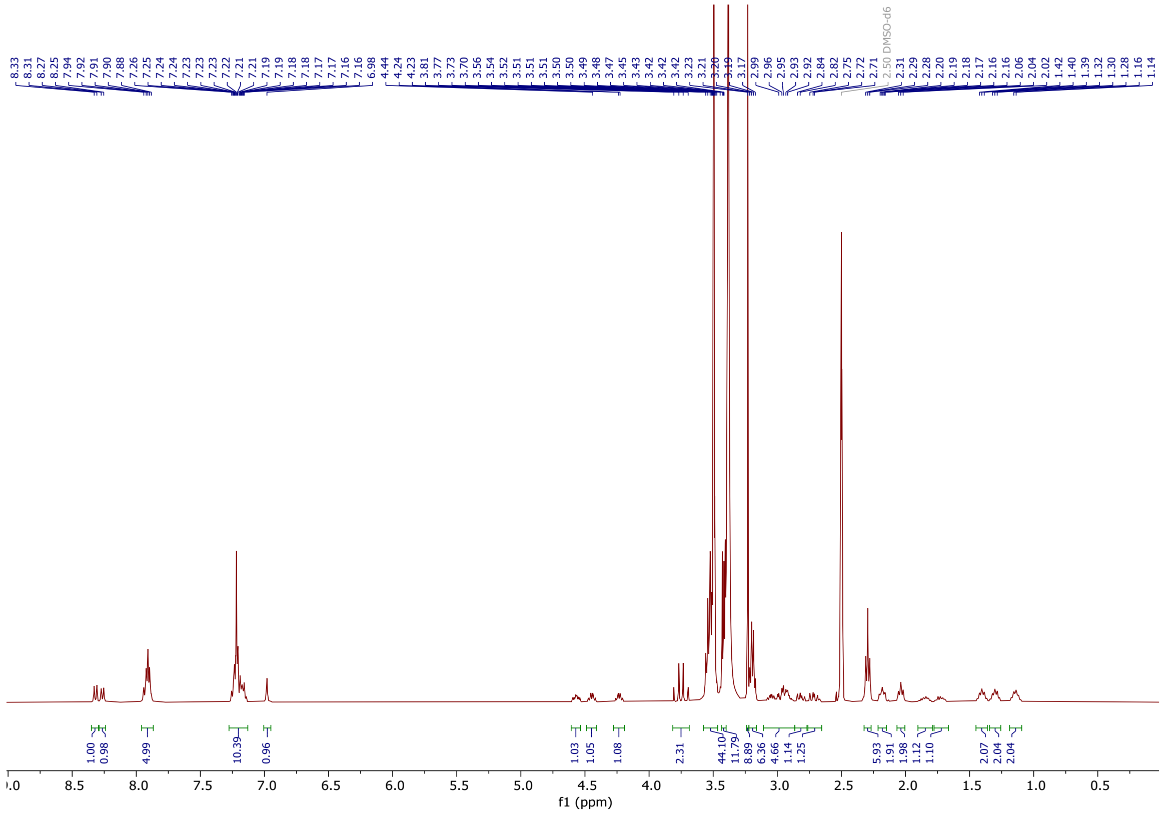

Figure S20: ^{1}H NMR spectrum of compound **4** in DMSO-d_6.

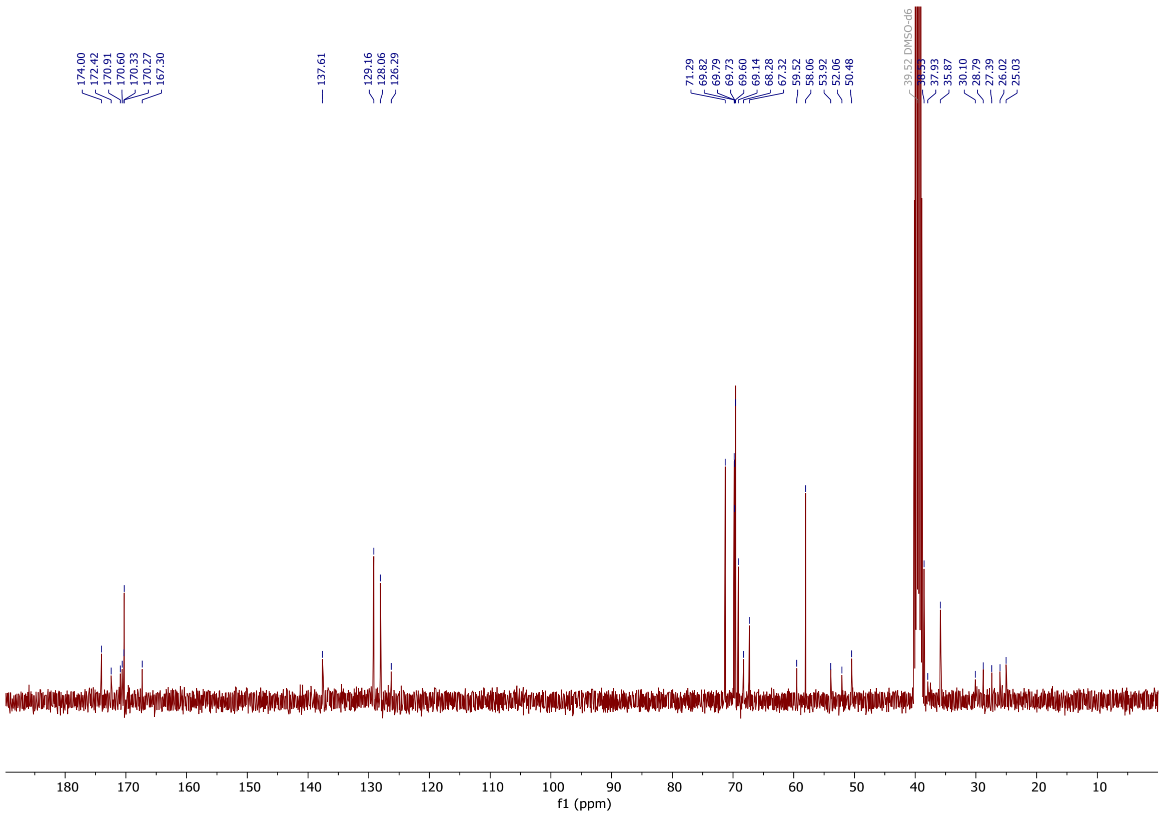

Figure S21: ^{13}C NMR spectrum of compound **4** in DMSO-d_6.

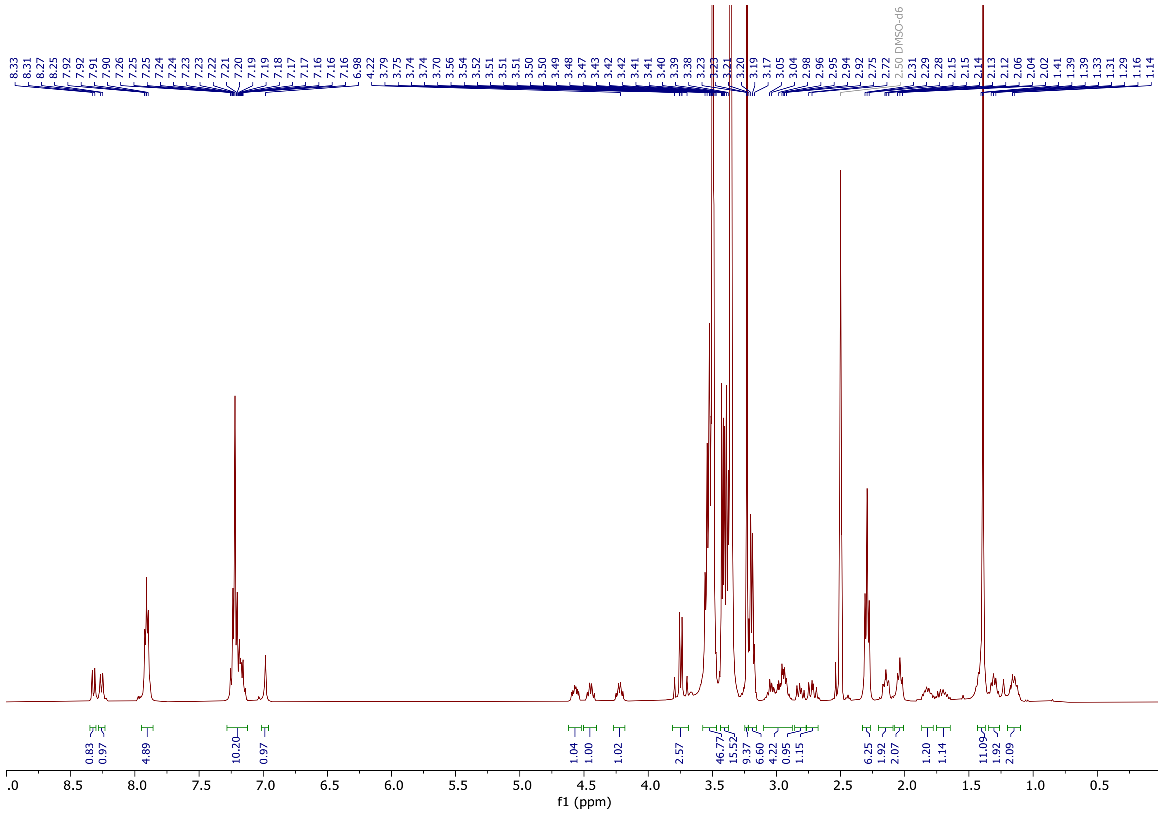

Figure S22: ^{1}H NMR spectrum of compound **5** in DMSO-d_6.

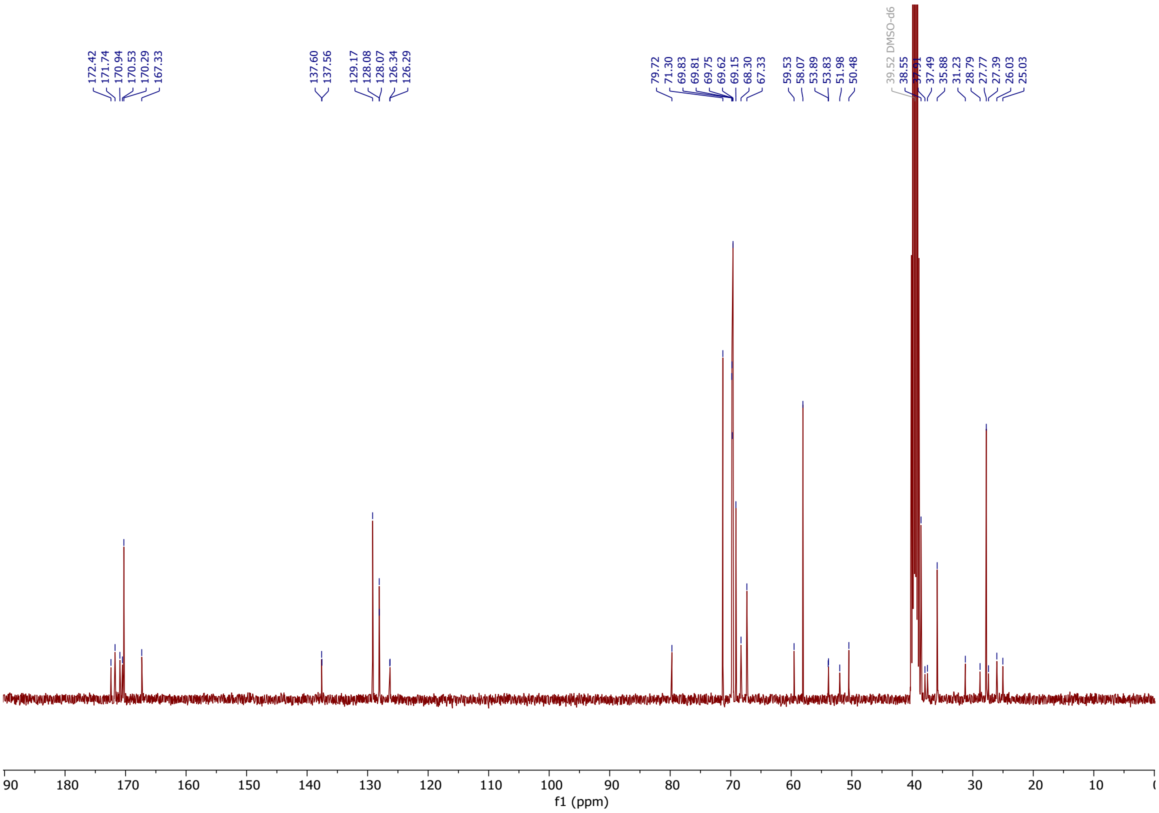

Figure S23: ^{13}C NMR spectrum of compound **5** in DMSO-d_6.

5 References

[1] H. E. Gottlieb, V. Kotlyar, A. Nudelman, *J. Org. Chem* **1997**, *62*, 7512–7515.

[2] E. Atherton, H. Fox, D. Harkiss, C. J. Logan, R. C. Sheppard, B. J. Williams, *J. Chem. Soc. Chem. Commun.* **1978**, 537–539.

[3] V. Lewe, M. Preuss, E. A. Woźnica, D. Spitzer, R. Otter, P. Besenius, *Chem. Commun.* **2018**, *54*, 9498–9501.

[4] D. Spitzer, L. L. Rodrigues, D. Straßburger, M. Mezger, P. Besenius, *Angew. Chemie Int. Ed.* **2017**, *56*, 15461–15465.
